# Unraveling the Nuclearity Effect of Atomically Choreographed Triatom Cu_3_ Clusters Supported on Zeolites

**DOI:** 10.1021/jacs.5c02706

**Published:** 2025-05-08

**Authors:** Tianxiang Chen, Yunong Li, Ping-Luen Ho, Kwan Chee Leung, Jinjie Liu, Ching Kit Tommy Wun, Zehao Li, Chiu Chung Tang, Shogo Kawaguchi, Tai-Sing Wu, Yun-Liang Soo, Jun Yin, Shik Chi Edman Tsang, Tsz Woon Benedict Lo

**Affiliations:** † State Key Laboratory of Chemical Biology and Drug Discovery, Department of Applied Biology and Chemical Technology, The Hong Kong Polytechnic University, Hung Hom, Kowloon 516083, Hong Kong, China; ‡ The Hong Kong Polytechnic University Shenzhen Research Institute, The Hong Kong Polytechnic University, Shenzhen 518057, China; § Inorganic Chemistry Laboratory, Department of Chemistry, University of Oxford, Oxford OX1 3QR, United Kingdom; ∥ Department of Materials, University of Oxford, Oxford OX1 3PH, United Kingdom; ⊥ Department of Applied Physics, The Hong Kong Polytechnic University, Hung Hom, Kowloon 999077, Hong Kong, China; # School of Chemistry and Chemical Engineering, Anyang Normal University, Anyang 455000, China; ∇ Diamond Light Source Ltd., Harwell Science and Innovation Campus, Didcot, Oxfordshire, Harwell Campus, Oxford OX11 0DE, United Kingdom; ○ Japan Synchrotron Radiation Research Institute (JASRI), SPring-8, 1-1-1 Kouto, Sayocho, Sayo-gun, Hyogo 679-5198, Japan; ◆ National Synchrotron Radiation Research Center, 101 Hsin-Ann Road, Hsinchu 30076, Taiwan; ¶ Department of Physics, National Tsing Hua University, Hsinchu 30013, Taiwan; †† PolyU-Daya Bay Technology and Innovation Research Institute, The Hong Kong Polytechnic University, Huizhou 510960, China

## Abstract

A precise understanding of the structure–activity relationship of catalysts is crucial for catalysis research and is essential for rationalizing next-generation catalysts. As the size of catalysts decreases from nanometric to atomic dimensions, the focus on structure–activity relationship correlation has shifted from the “size effect” to the much more challenging “metal nuclearity effect”. However, precise synthesis and reliable characterization for structurally related solid atomic catalysts, such as single-, dual-, and triatom catalysts, still remain extremely challenging. Here, we present the controlled assembly of single-atomic Cu_1_, dual-atomic Cu_2_, and triatomic Cu_3_ supported on zeolites through an innovative atomically choreographed approach. For the first time, we have directly visualized the atomic features of Cu_3_ with respect to the zeolitic channels using double aberration-corrected scanning transmission electron microscopy (STEM). The structural and electronic properties of the catalysts have been characterized using synchrotron X-ray absorption spectroscopy, high-resolution synchrotron powder X-ray diffraction (PXRD), and density functional theory (DFT) calculations. We revealed the interplay among surface structures, adsorption configurations, catalytic reactivities (showing a significant 25-fold improvement), and product selectivity across structurally related species using a model methanol reforming reaction. We have successfully elucidated the relationship between the metal nuclearity effect and its activity and selectivity in a complex catalytic reaction. Our findings offer an unprecedented opportunity for the catalysis and materials community to finely manipulate the physicochemical properties of this category of solid atomic catalysts to achieve the desired reactivities and selectivities.

## Graphical abstract

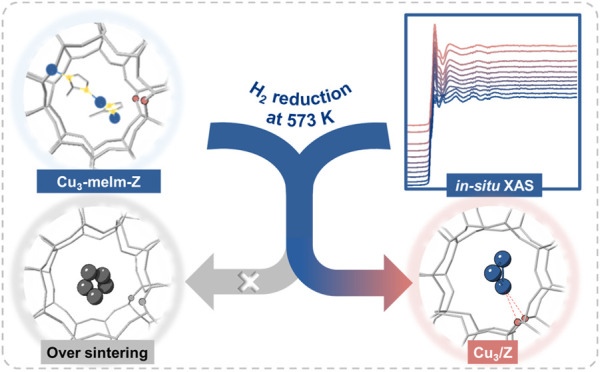

## Introduction

Understanding the structure–activity relationship is pivotal to developing next-generation catalysts. Previous studies have extensively investigated the “size effect”, revealing the dependence of reactivity and selectivity of catalysts on the size of the nanoparticles (Scheme 1a).^1–3^ However, conventional studies on “size effects” do not provide sufficient insights from atomic and molecular perspectives.^4–7^ This gap is particularly significant given the increasing volume of research focused on solid-state catalysts in the “atomic” regime, such as supported single-atom catalysts and supported clusters with low nuclearity (*n* < 5). These solid atomic catalysts are demonstrating great potential in catalytic applications, particularly in energy production and fine chemical synthesis. As the size decreases from a few nanometers to subnanometric dimensions, surface atoms become increasingly dominant, and the atomic configurations become vital, as they are highly sensitive to incoming reaction substrate(s). Notably, the chemical and physical properties of catalysts change drastically when their size is reduced to less than 1 nm, directly depending on the number of atoms comprising the active site—the “metal nuclearity effect”.^8^ More specifically, the changes in metal nuclearity directly influence the electronic structure, coordination environment, and geometric configuration of the active site. They directly affect the adsorption and activation of reaction substrates as well as the stabilization of specific reaction intermediates/transition states. To our delight, recent advances in synthesis methods and characterization techniques have enabled researchers to more extensively and systematically explore these solid atomic catalysts.^9^

**Scheme 1:**
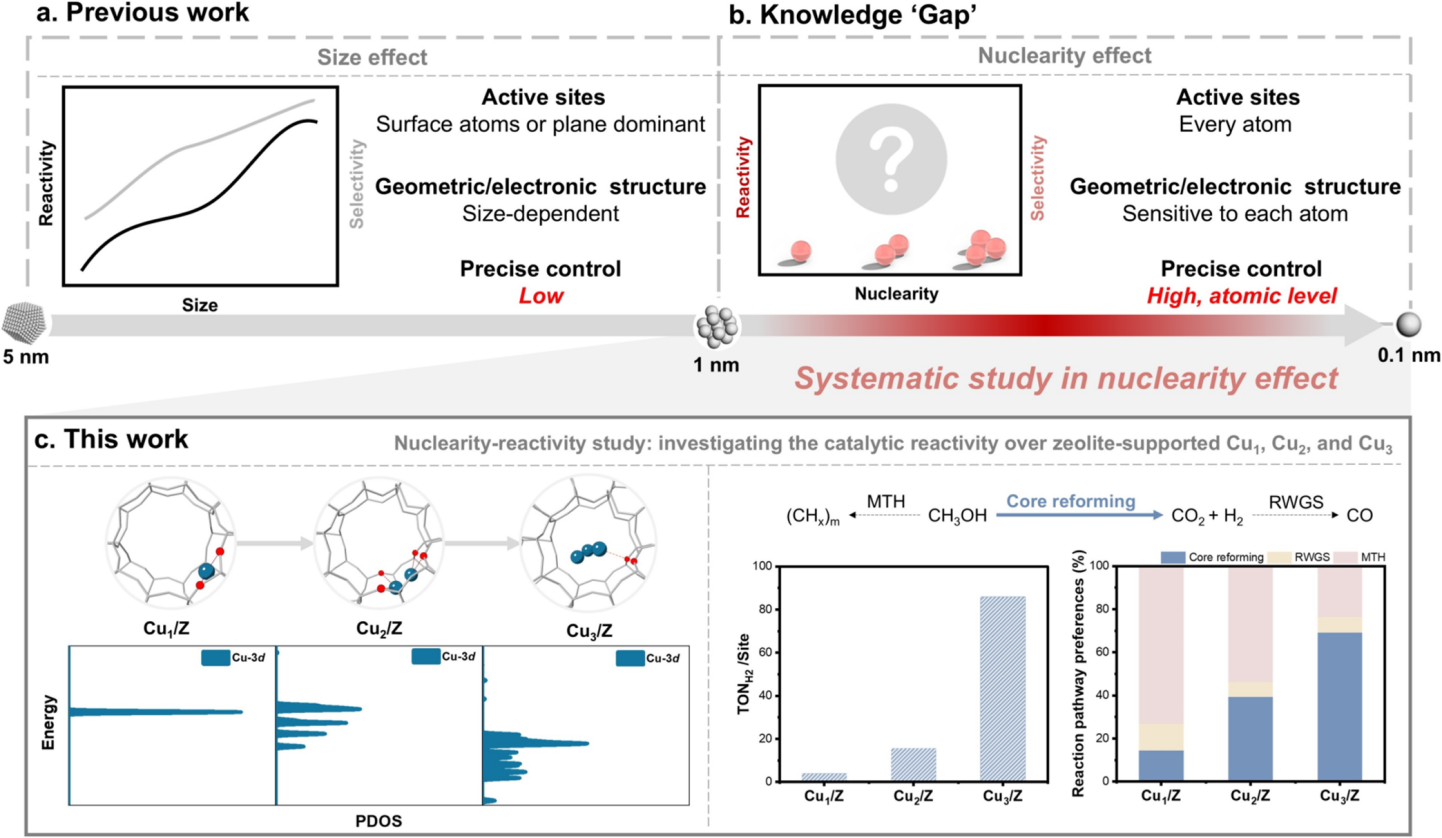
(a) Structure–Activity Relationship Study Focusing on the “Size Effect”. (b) Knowledge Gap in the “Nuclearity-Reactivity” Science. (c) Systematic Study of the Nuclearity Effect of Atomically Choreographed Cu_*x*_/Z Using Methanol Reforming as a Model Reaction

The development of supported single-atom catalysts has successfully brought heterogeneous catalysts to the atomic level, which typically exhibit high selectivity due to their uniform active sites.^10–13^ Supported clusters with low-nuclearity, immediate variants of supported single-atom catalysts have emerged as a promising class of catalytic materials.^14–19^ Theoretically, they can effectively bridge the gap between classical bulk nanomaterials and atomic/molecular species, advancing supported catalysts toward the molecular frontier.^20^ The neighboring atoms in these supported clusters could introduce synergistic effects between neighboring atoms, enabling more complex reaction pathways or enhanced activity.^17,21,22^ However, the precise synthesis and hence systematic “nuclearity-reactivity” investigation of supported well-defined metal clusters with low-nuclearity remains challenging (Scheme 1b). Herein, we present a modified bottom-up method for developing well-defined single-atomic Cu_1_, dual-atomic Cu_2_, and triatomic Cu_3_ supported on zeolites [Cu_*x*_/Z (*x* = 1, 2, and 3)] (Scheme 1c).^16,21,23^ The structural characteristics and electronic properties have been revealed by combining with quantitative analysis of synchrotron X-ray absorption spectroscopy (XAS), Rietveld refinement of high-resolution synchrotron X-ray powder diffraction (PXRD) analysis, and density functional theory (DFT) calculation. For the first time, we have directly visualized the atomistic features of the homometallic Cu_3_/Z triatomic catalysts (TACs) using double aberration-corrected scanning transmission electron microscopy (STEM). Accordingly, we have systematically investigated the nuclearity effect of these atomically choreographed Cu_*x*_/Z (*x* = 1, 2, and 3) over the methanol reforming reaction, where a series of competing reactions are present. In this chemical probe reaction, extensively tunable catalytic properties and inhibition of competing reaction pathways have been observed in which the Cu_3_ TACs exhibit unparalleled reactivity. More specifically, Cu_3_/Z achieves a 25-fold increase in H_2_ yield and demonstrates a notably higher selectivity when compared with its “single-atomic” Cu_1_/Z counterpart.

## Results and Discussion

For this study, a highly crystalline H-ZSM-5 zeolite with a moderate distribution of Brønsted acid sites (BASs) was selected. The H-ZSM-5 has a SiO_2_/Al_2_O_3_ ratio of 46 and possesses a homogeneous distribution of Al at the crystallographic T6 site, with approximately 4 Al per unit cell, as previously characterized by our team.^24–26^ This characteristic helps to prevent metal aggregation and facilitates reliable structural investigations. Initially, Cu^2+^ ions were incorporated into the micropore of H-ZSM-5 through a simple ion-exchange process with the existing BASs, resulting in Cu_1_-ZSM-5 (referred to as “Cu_1_-Z”). After thorough washing, the anchored Cu^2+^ site served as the coordination site for the incoming dibasic 2-methylimidazole molecule (“meIm”), forming a local “–Cu-meIm” complex. The remaining pyridinic (–N=) site was then deprotonated to coordinate with the next Cu^2+^ species through another “ion-exchange” step, resulting in the formation of a supported binuclear “–Cu-meIm-Cu” complex (“Cu_2_-meIm-Z”). By repeating the coordination step with meIm and the metalation step with Cu^2+^, a trinuclear Cu_3_ complex supported on zeolite (“Cu_3_-meIm-Z”) is prepared. At this stage, Cu_3_-meIm-Z should exist in the form of supported metal complexes. To remove the meIm linkers completely, a critical pretreatment step was undertaken.

To determine a suitable hydrogen reduction pretreatment condition that precisely assembles metal ions together, we employed a series of *in situ* characterization techniques. Figure 1a,b shows the results of *in situ* extended X-ray absorption fine structure spectroscopy of Cu_2_-meIm-Z and Cu_3_-meIm-Z precursor in flowing hydrogen (H_2_-EXAFS). The data reveal apparent changes in the coordination environment and overall structural characteristics of the copper species. Given the similarity in the *in situ* EXAFS profiles between Cu_2_-meIm-Z and Cu_3_-meIm-Z, our discussion will focus on the Cu_3_-meIm-Z data set to ensure conciseness. At temperatures below 573 K, the radial distance distribution is primarily centered around *r* ∼ 1.5 Å, corresponding to the backscattering of Cu–N/O bonds. Notably, no Cu–Cu backscattering is observed at temperatures below 573 K, suggesting that copper exists as a meIm-mediated copper complex. As the temperature increases, the peak intensity gradually decreases, indicating the removal of meIm and water ligands (as confirmed by ultraviolet–visible (UV–vis) spectroscopy and thermogravimetric analysis, shown in Figures S1–S2 in the Supporting Information (SI)). At 573 K, a peak emerges at *r* ∼ 2.2 Å, indicating the gradual formation of Cu–Cu bonds. At temperatures above 601 K, apparent copper sintering occurs, as evidenced by the increase in the Cu–Cu coordination number (CN) from ∼2 to ∼6 (Figure 1c–d). Specifically, at a reduction temperature of 573 K, the average CNs of Cu–O and Cu–Cu are determined to be 0.9(1) and 2.1(1), respectively, indicating the presence of supported Cu_3_ species on the internal surface of H-ZSM-5. A comprehensive quantitative fitting analysis is provided in Figures S3 and S5 and Tables S1–S2.

**Figure 1:**
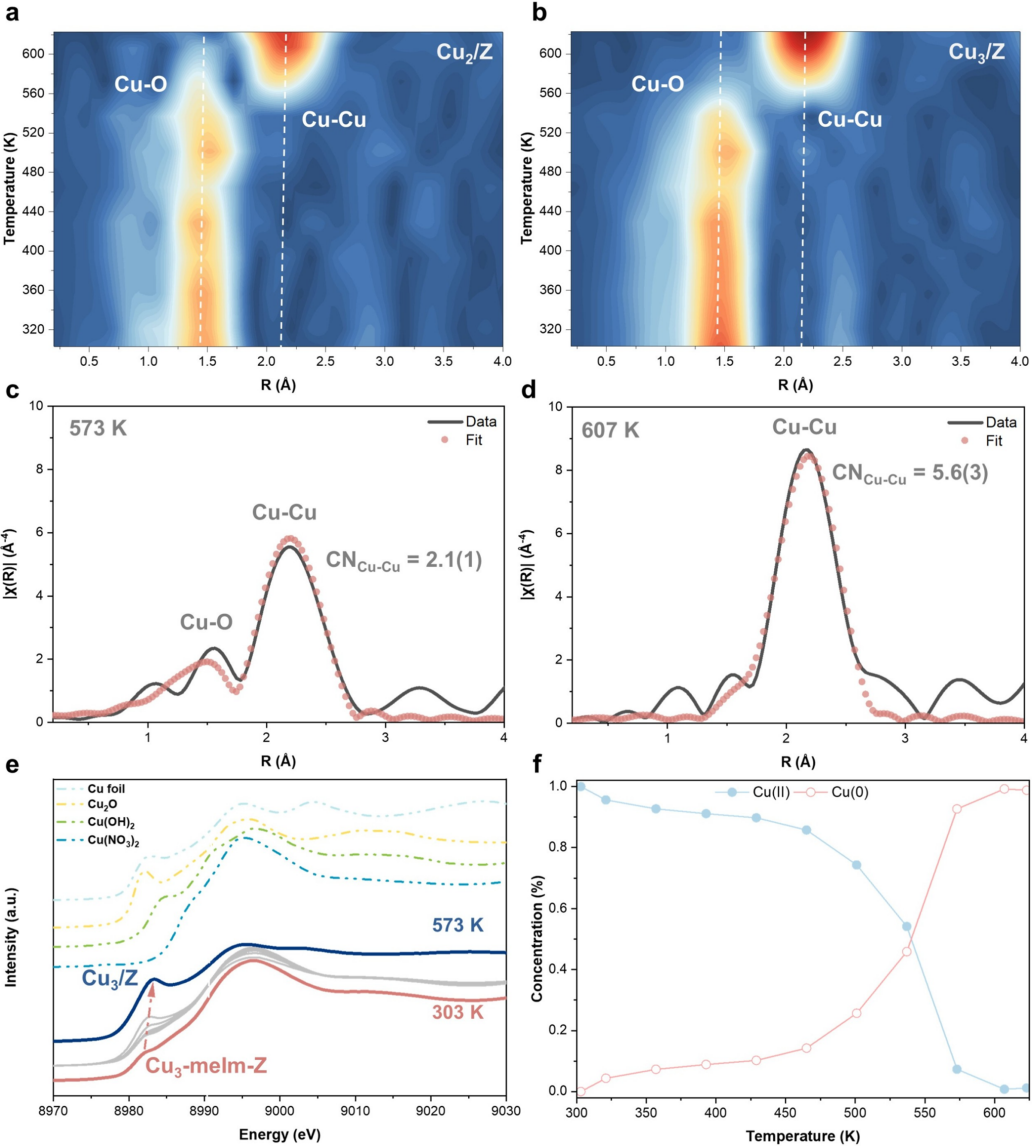
Determination of ligand removal condition. *In situ* EXAFS of the (a) Cu_2_-meIm-Z and (b) Cu_3_-meIm-Z precursor under a hydrogen atmosphere. Cu K-edge EXAFS (black) and fitting (red) are shown in *k* ^3^ weighted *R*-space of Cu_3_-meIm-Z reduced at (c) 573 K and (d) 607 K. (e) *In situ* XANES of the Cu_3_-meIm-Z under a hydrogen atmosphere and (f) dynamic evolution of the oxidation state of the Cu species obtained by MCR-ALS analysis of the *in situ* XANES data set.

The reduction of copper species and the formation of Cu–Cu bonds are also supported by various characterization techniques, including X-ray photoelectron spectroscopy (XPS) and Auger electron spectroscopy (AES) analysis (Figures S6–S7 and Table S3), temperature-programmed reduction analysis under a hydrogen environment (H_2_-TPR, Figure S8), thermogravimetric analysis under hydrogen (H_2_-TGA, Figure S9), and *in situ* X-ray absorption near-edge spectroscopy under hydrogen (H_2_-XANES, Figure 1e–f). XPS analysis confirms the reduction process by showing a shift in the Cu 2p peak and the emergence of a Cu(0) peak. H_2_-TPR analysis demonstrates consistent reduction behavior, with a peak observed at around 570 K, indicating copper reduction. H_2_-TGA investigates the chemical changes during the removal of meIm linkers, with the predominant removal occurring above 550 K. We therefore determined that a pretreatment temperature of 573 K is suitable for producing a meIm-free “Cu_2_/Z” and “Cu_3_/Z” catalyst from the corresponding precursor. Principal component analysis (PCA) on the *in situ* XANES data set was employed to reduce the dimensionality of the electronic structure data for Cu_3_/Z. The first two principal components captured nearly all experimental (*e.g.*, >99.95%; see Figure S11), indicating that the data set’s complexity could be simplified into two dominant spectral features. Subsequent multivariate curve resolution by alternating least-squares (MCR-ALS) analysis decomposed these components into two distinct spectral profiles: one resembling CuO (Cu^2+^) and the other metallic Cu (Cu^0^) (Figures 1f and S11). At 573 K, the Cu(0):Cu­(II) ratio (0.927:0.073) confirmed a mixed Cu^δ+^ electronic configuration (0 < δ < 2), yielding an average oxidation state of ∼+0.5 for Cu_3_/Z.

The copper contents of the meIm-free samples, *i*.*e*., Cu_1_/Z, Cu_2_/Z, and Cu_3_/Z, increase from 1.13 to 2.12 to 3.70 wt %, respectively, as summarized in Figure S10 and Table S4. This corresponds to atomic Cu/Al molar ratios of 0.30, 0.56, and 0.97, indicating controlled and successive addition of copper species. In a control experiment without the addition of the meIm linker, the copper content remained relatively unchanged at around 1.20 wt %. Taken together, the collective findings provide consistent evidence for copper reduction, removal of meIm linkers, and the formation of Cu–Cu bonds in diatomic Cu_2_/Z and triatomic Cu_3_/Z.

We subsequently employed synchrotron PXRD to study the crystallographic properties of these samples. Utilizing an optimized synchrotron X-ray at an energy of 15 keV (λ = 0.824861(2) Å), we observed distinct differences in the Bragg intensities in the synchrotron PXRD data among the related samples (Figure S12). Due to the significant electron density contrast between the Cu atoms and the Si and O framework atoms, we initially employed the difference Fourier charge flipping algorithm to determine the crystallographic positions and concentrations of the extra-framework Cu metal nuclei (Figure S13). To further investigate the locations of the extra-framework species, we conducted Rietveld refinement using TOPAS V7.0 academic software, revealing the corresponding atomic parameters of Cu_*x*_/Z (*x* = 1, 2, and 3) (Figure 2a–f and Tables S5–S8) as well as their spatial relationship with respect to the ZSM-5 framework. As presented in the Rietveld refined crystal structure (Figure 2d), Cu_1_/Z possesses a mononuclear Cu site at the sinusoidal channel within a distance of 3.03 Å between the Cu atom and framework O18. In Cu_2_/Z, we observed two distinct crystallographic sites, each containing a Cu_2_ species. Site A is located at the straight-sinusoidal channel, whereas Site B is located at the sinusoidal channel. These two sites are separated by more than 5 Å, indicating that they do not form a large cluster but rather statistically coexist as Cu_2_ in different asymmetric units. These Cu–Cu bond distances measure approximately 2.3 Å. As depicted in Figure 2f, Cu_3_/Z also displays two assembled triangular Cu_3_ sites as determined by Rietveld refinement, with Cu_I_–Cu_III_ (Site A) at straight-sinusoidal channel intersection and Cu_IV_–Cu_VI_ (Site B) within the sinusoidal channel. The Cu–Cu distances between these Cu_3_ species measure approximately 2.25**–**2.36 Å, aligning with the quantitative analysis derived from our *in situ* H_2_-EXAFS analysis. Density functional theory (DFT) calculations were conducted to gain a deeper understanding of the interaction between Cu_3_ and zeolite supports (Figure 2g). The optimized Cu_3_/Z structure reveals the presence of an approximately diamagnetic Cu_3_^+^ triangle with Cu–Cu bond lengths ranging from 2.25 to 2.41 Å. The Cu_3_^+^ species is anchored to oxygen atoms within the zeolite cavity, forming Cu–O_framework_ bonds at approximately 2.1 Å. One copper atom within the cluster remains uncoordinated. The calculated near-zero spin densities indicate an electron transfer from the Cu_3_ species to the zeolite framework, resulting in an overall Cu_3_^+^ state. These DFT results are consistent with the experimental observations of XANES, further affirming the findings. We also employed synchrotron X-ray total scattering to study the structural features of Cu_3_/Z (*E* = 60 keV, λ = 0.206641 Å, *Q*_maxinst_ = 30 Å^–1^). The X-ray pair distribution function data of Cu_3_/Z exhibit a similar long-range oscillating structure to the ZSM-5 support. The short-range variations (particularly notable within *R* < 5 Å) originate from the Cu_3_ cluster and its interaction with the zeolite framework (Figure 2h). A combined analysis of the synchrotron X-ray pair distribution function and Rietveld refinement was conducted to further optimize the atomic model (Figure 2i–j).

**Figure 2:**
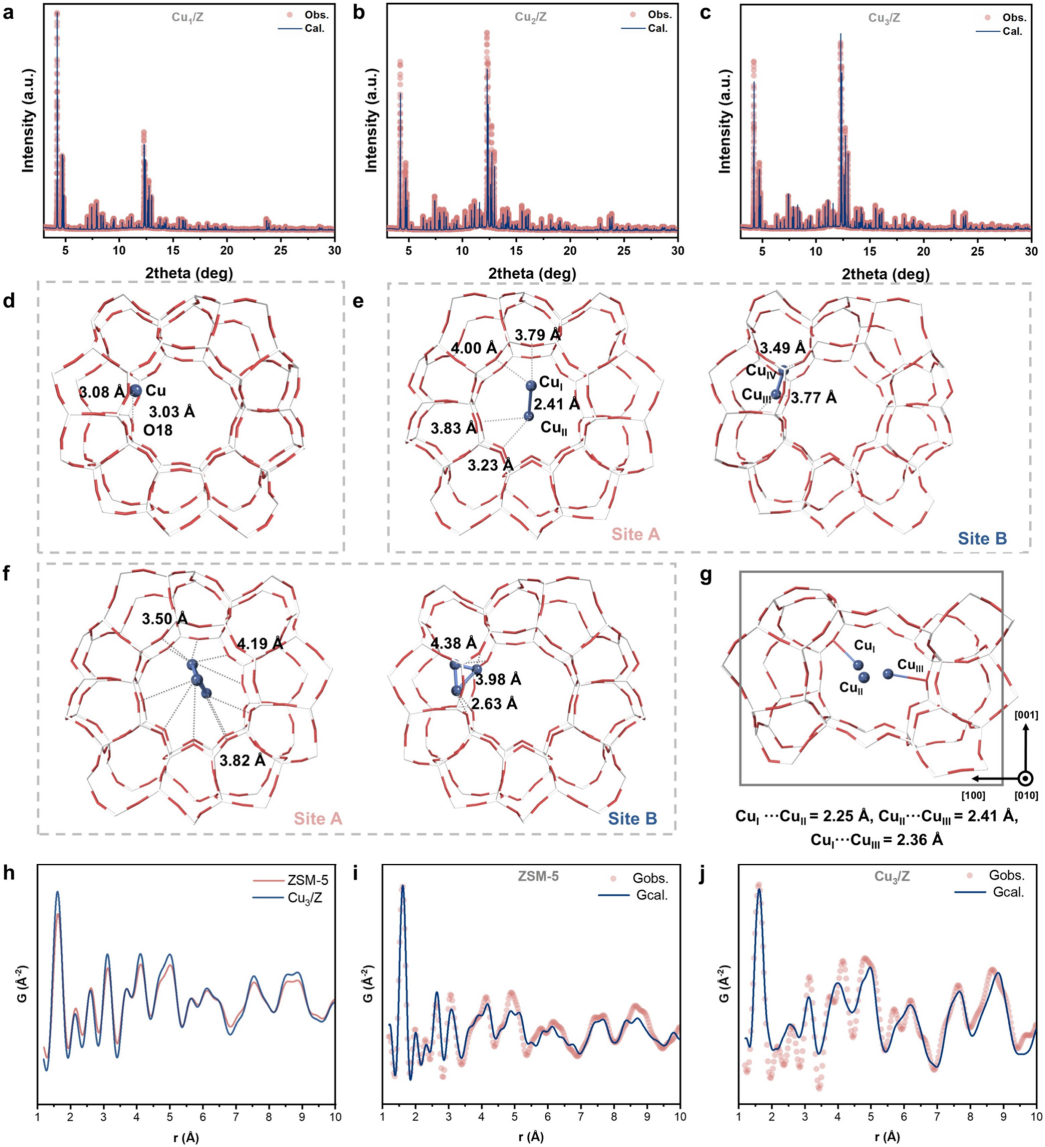
Structure determination of Cu_3_/Z. Rietveld refinement profiles of high-resolution synchrotron PXRD of (a) Cu_1_/Z, (b) Cu_2_/Z, and (c) Cu_3_/Z. High-resolution synchrotron PXRD patterns were collected on beamline I11 at the diamond light source (λ = 0.824681 Å; *E* = 15 keV) and the Rietveld refinement profiles using TOPAS V7.0. The corresponding Rietveld refined crystal structures are (d) Cu_1_/Z, (e) Cu_2_/Z, and (f) Cu_3_/Z. (g) Optimized crystal structure of Cu_3_/Z obtained by plane-wave DFT calculation. (h) X-ray pair distribution function analysis of pristine H-ZSM-5 zeolite and Cu_3_/Z, and the corresponding combined fitting profiles of (i) H-ZSM-5 and (j) Cu_3_/Z.

Building on these structural findings obtained from synchrotron X-ray techniques, we further employed double aberration-corrected scanning transmission electron microscopy (STEM) to investigate the electronic properties and confirm the presence of extra-framework copper species within the zeolite framework. In our study, we first utilized electron energy loss spectroscopy (EELS) to investigate whether the signal contrast of interest originates from the copper atoms (Figure S14). The broad L_2_ and L_3_ edges observed suggest a predominant presence of Cu(0) in Cu_3_/Z.^27^ The identity of the copper species has been confirmed by comparing the image contrast between pristine H-ZSM-5 and Cu_3_/Z. Importantly, the pristine H-ZSM-5 exhibits no significant contrast of superdiffraction lattices when comparing equivalent crystallographic directions (Figure 3a). High-angle annular dark-field (HAADF) images (Figure 3b–f) provided further insights, revealing two distinct Cu_3_ species along the [010] straight channel of the ZSM-5 framework. The pink and blue boxes indicate the locations of the Cu_3_ species: Site A is situated in the straight-sinusoidal channel intersection, while Site B is positioned within the sinusoidal channels. Remarkably, the strong agreement between the experimental observations from HAADF-STEM and the refined crystal structure obtained above enables a clear understanding of the atomic structure of the two Cu_3_ sites within the zeolite support (Figure 3g–h).

**Figure 3:**
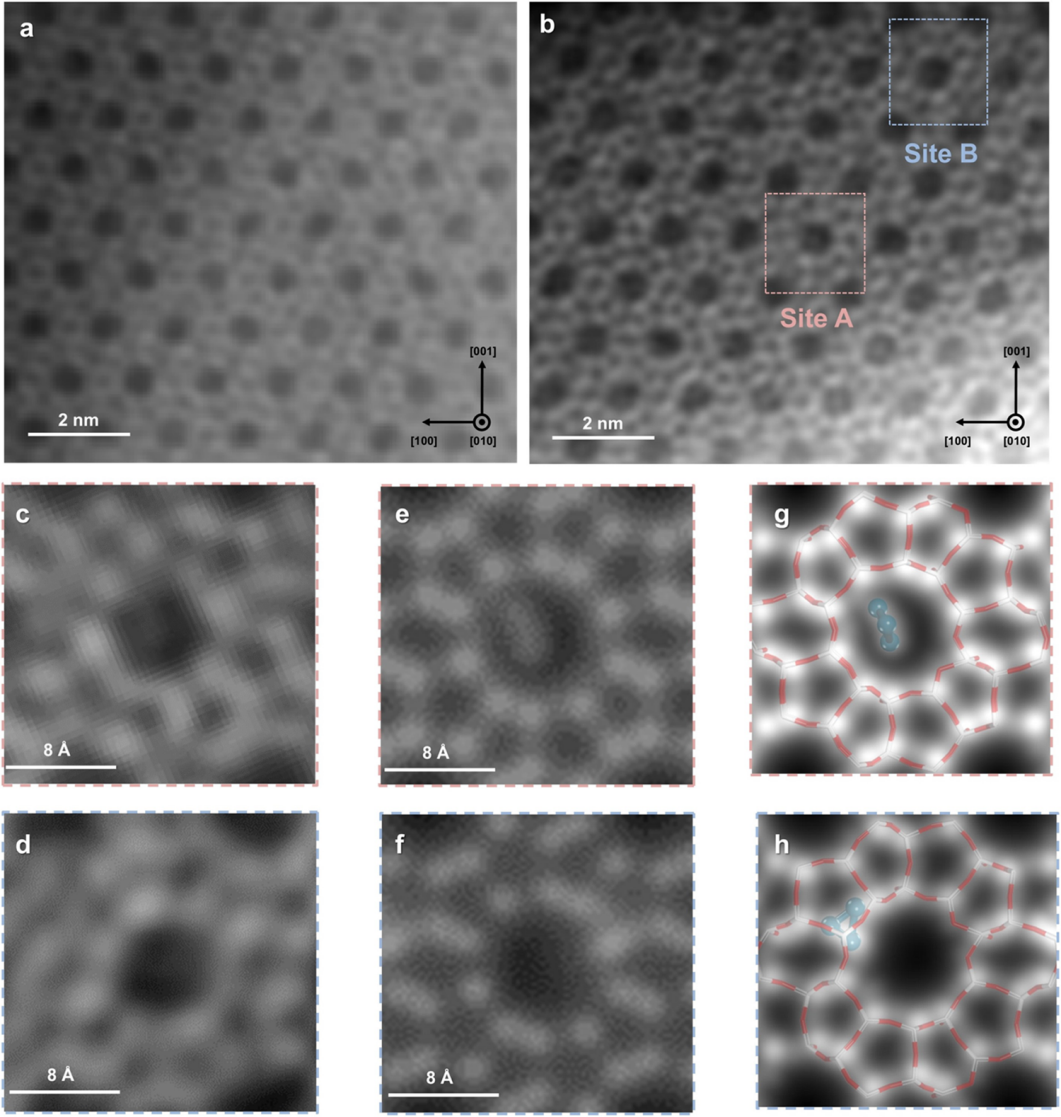
HAADF-STEM image of (a) pristine H-ZSM-5 and (b) Cu_3_/Z along the [010] zone axis. Enlarged HAADF-STEM images of (c) Site A, (d) Site B, and (e, f) the corresponding simulated images, respectively. Comparison between the refined crystal structure obtained from synchrotron PXRD and the corresponding HAADF-STEM images of (g) Site A and (h) Site B. The mirror symmetry of the extra-framework species has been disregarded for clarity. Atoms are represented in balls/sticks (dark blue = copper, white = silicon, and red = oxygen).

To understand the electronic communication among the Cu species, we further compared the projected partial density of states (PDOS) of Cu_3_ TACs with that of mononuclear Cu_1_ (Figure S15). The resulting d-band center for Cu_1_/Z is located at −0.47 eV (*E*_F_ = 0). With an increase in metal nuclearity, the d-band centers demonstrate a decreasing trend: −0.71 eV for Cu_2_/Z and −1.98 eV for Cu_3_/Z. This suggests a significant distinction in the electronic characteristics of Cu_3_/Z compared to Cu_2_/Z and Cu_1_/Z. The observed notable energy splitting in the Cu 3d fine structure can be attributed to the effective overlap between the Cu 3d orbitals at the bonding distance. These energy splittings, along with the downshift in the d-band center and modifications in the band-splitting structure, have the potential to greatly alter the binding interactions between the Cu species and incoming substrate molecules. This, in turn, can have a notable impact on the catalytic properties and reactivity.^28,29^

## Studying the “Metal Nuclearity” Effect

Integration of microscopic, crystallographic, and spectroscopic characterization has yielded a comprehensive understanding of the atomic and structural properties of the Cu species within ZSM-5 zeolites. Building upon this knowledge, we first proceeded to investigate the impact of “metal nuclearity” on catalysis by examining the catalytic properties of Cu_1_/Z, Cu_2_/Z, and Cu_3_/Z in the methanol reforming reaction (MeOH + H_2_O → CO_2_ + H_2_). This specific reaction is highly influenced by the electronic and steric properties of the active sites, particularly in terms of their preference for activating C–H versus O–H bonds. The efficiency and selectivity of the reaction depend significantly on this preference.^30^ The methanol reforming reaction should hence serve as an effective model to explore the reactivity descriptors due to the presence of multiple competing reaction pathways, including (a) core reforming (yielding CO_2_ and H_2_), subsequent (b) reverse water–gas shift (RWGS, yielding CO and H_2_O), and (c) methanol to hydrocarbons (MTH).^31^

The catalytic performance in the methanol reforming reaction was evaluated using a batch reactor (Figure S16). Experimental analysis of the methanol reforming reaction revealed the formation of various gaseous products including H_2_, CO, CO_2_, and C_1_–C_4_+ hydrocarbons. To optimize the reaction conditions and enhance selectivity, factors such as reaction temperatures (Figure S17) and methanol-to-water ratio (Figure S18) were carefully examined. The catalytic performance of pristine H-ZSM-5 and Cu_*x*_/Z catalysts is presented in Figure 4a. H-ZSM-5 and Cu_1_/Z exhibited low H_2_ yield of 203.3 and 698.4 μmol g_cat_^–1^, respectively, whereas Cu_2_/Z and Cu_3_/Z exhibited significantly higher H_2_ yield, with 2614.4 and 16229.5 μmol g_cat_^–1^, respectively. Conversely, H-ZSM-5 and Cu_1_/Z exhibit much higher product distributions of CO and C_1_–C_4_+ hydrocarbons when compared with Cu_2_/Z and Cu_3_/Z. For a fairer comparison, the turnover number (TON, calibrated per “site”) for methanol reforming for Cu_3_/Z was more than 25-fold that of Cu_1_/Z and 5-fold that of Cu_2_/Z (Figure 4b). This indicates a shift in preference for the core reforming pathway in the Cu_3_/Z catalyst, increasing from 14.4% for the single-atom Cu_1_/Z catalyst to 69.1%—approximately a 5-fold increase. In contrast, the competing RWGS and MTH pathways were much preferred in H-ZSM-5 and Cu_1_/Z (Figure 4c). It is noted that the MTH pathway is known to be favorably catalyzed by zeolitic BASs, as evidenced by the almost exclusive selectivity for C_2_–C_4_+ hydrocarbons over H-ZSM-5. Furthermore, the H_2_:CO_2_ ratio over Cu_3_/Z is at 2.76, much closer to the ideal value of 3:1 if the core reforming pathway is the only reaction (*cf*. 1.50 in Cu_1_/Z and 2.11 in Cu_2_/Z). This ratio indicates the preference for the methanol-reforming pathway, while inhibiting the two other competing reactions. These findings highlight the superior catalytic performance of Cu_3_/Z compared to Cu_1_/Z and Cu_2_/Z in the methanol reforming reaction. We have also identified synergistic effects between the BAS-Cu_3_ pair in our poisoning experiment. In the poisoning experiment, most unoccupied BASs were blocked by introducing Na^+^ ions into ZSM-5 (Figure 4d). The comparison of catalytic activities between Cu_3_/Z and Cu_3_/Na-Z (where Na^+^ replaces H^+^) reveals that Cu_3_/Na-Z exhibits significantly inferior catalytic performance compared to Cu_3_/Z. The yield of CO_2_ and H_2_ is decreased by more than 70% in Cu_3_/Na-Z, indicating that the zeolitic BASs and Cu_3_ active sites work synergistically to enhance catalytic reactivity, which aligns with experimental catalytic studies.

**Figure 4:**
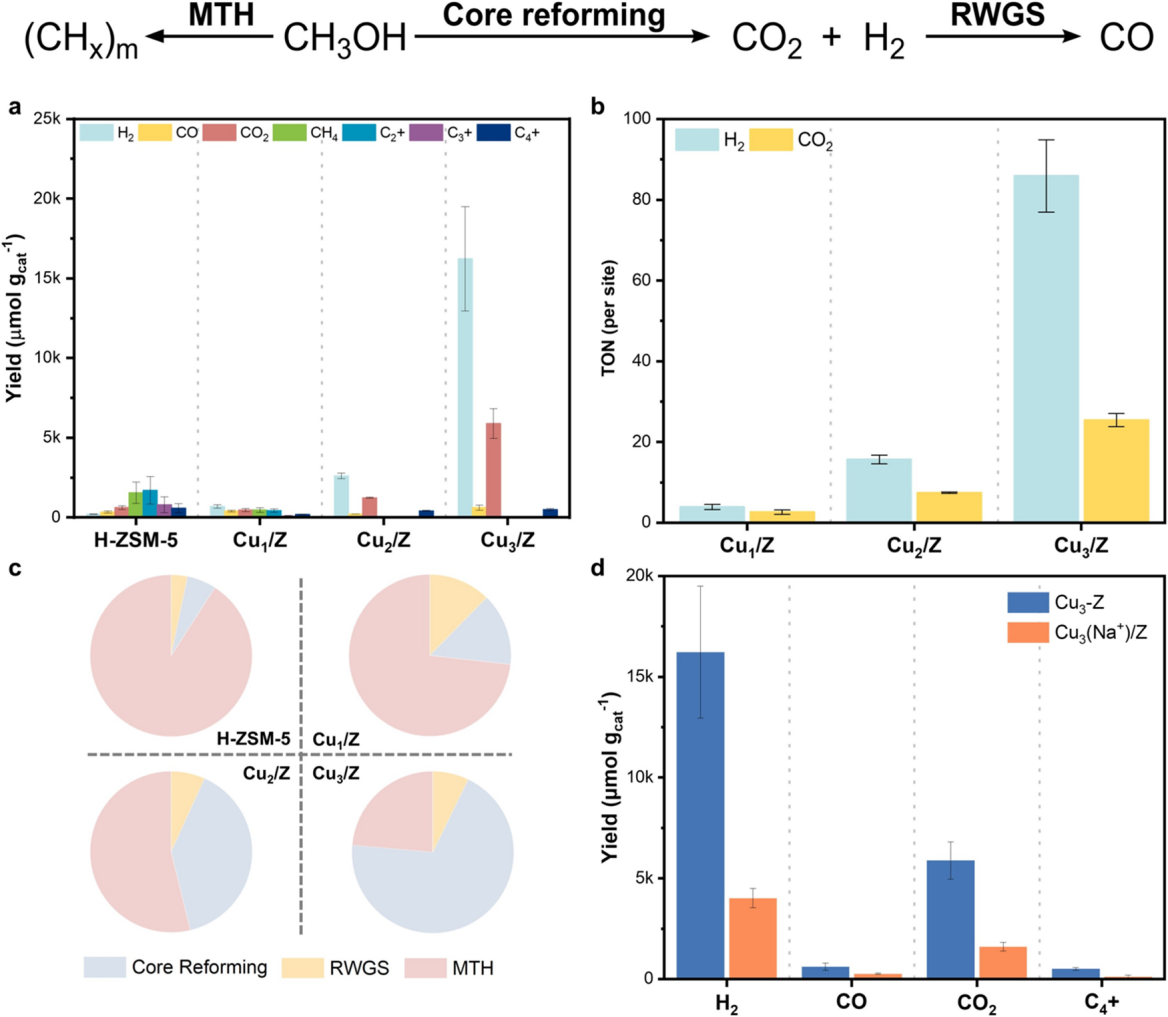
Catalytic performance evaluation. (a) Product distribution and yield in the methanol reforming reaction, (b) turnover numbers (TON) of Cu_*x*_/Z (*x* = 1, 2, and 3), (c) preference in the reaction pathway over pristine H-ZSM-5 zeolite, Cu_*x*_/Z (*x* = 1, 2, and 3), and (d) product distribution and yield in the methanol reforming reaction of Cu_3_/Z and Cu_3_/Z poisoned by Na^+^ (Cu_3_(Na^+^)/Z).

The comparison between the Cu_*x*_/Z catalysts reveals a clear “metal nuclearity” effect. To understand the underlying factors contributing to these differences, we investigated the adsorption behavior of methanol on the active sites of these catalysts, as it plays a crucial role in determining catalytic selectivity and reactivity.^32^ To gain insights into the adsorption behavior, we specifically conducted a comparative analysis using Cu_1_/Z and Cu_3_/Z. From our *in situ* diffuse reflectance infrared Fourier transform spectroscopy (DRIFTS) measurements, we detected the presence of Cu–O­(H) species at 3624 and 3611 cm^–1^ in both Cu_1_/Z and Cu_3_/Z (Figure 5a).^33^ However, the stretching vibration of Cu–O­(H)–CH_3_ was only observed in Cu_3_/Z but not in Cu_1_/Z. Furthermore, in the stretching region between 2600 and 3100 cm^–1^, we observed a gradual decrease in the intensity of asymmetric C–H (2948 cm^–1^) and symmetric C–H (2842 cm^–1^) stretches of methanol with increasing temperature (Figure 5b), suggesting C–H cleavage at elevated temperatures. However, at temperatures beyond 623 K, the C–H vibrations were only observed in Cu_3_/Z, indicating a stronger methanol binding on Cu_3_/Z compared to Cu_1_/Z.^34^ Clearly, we observed more favorable methanol adsorption on the Cu_3_ sites, which not only ensures further activation of methanol but also leads to the enrichment of active hydrogen species for the core reforming pathway.

**Figure 5:**
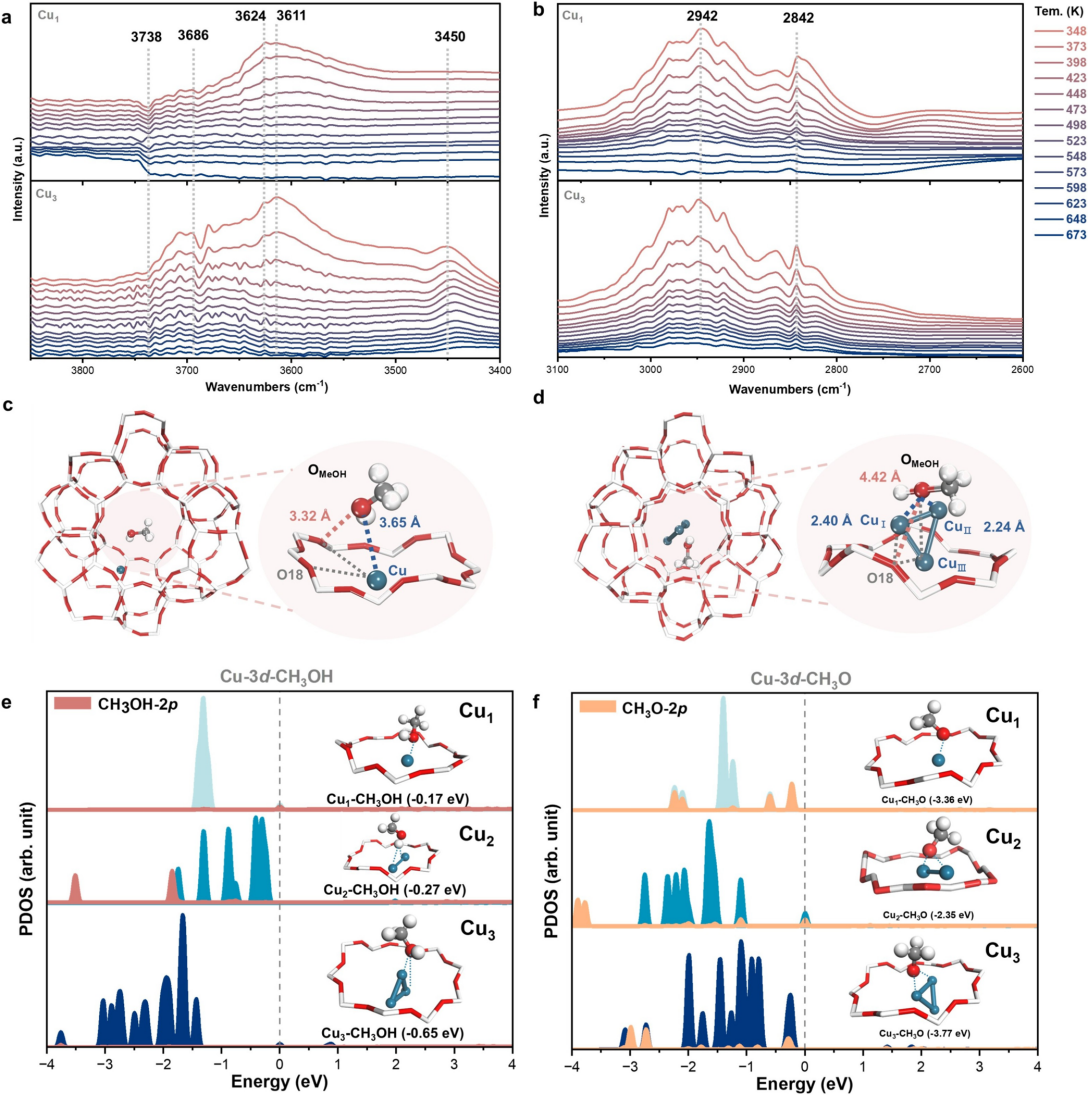
“Metal nuclearity” effect. (a, b) *In situ* DRIFTS spectra of Cu_3_/Z preadsorbed with methanol. The Rietveld refined structure of (c) Cu_1_/Z and (d) Cu_3_/Z (showing Site A) preadsorbed with methanol. The corresponding projected density of states (PDOS) of pristine ZSM-5 zeolite and Cu_*x*_/Z preadsorbed with (e) methanol (f) and CH_3_O*, respectively.

These insights into the adsorption characteristics prompted us to further employ Rietveld refinement to explore the geometries and interactions of methanol with the Cu sites, thereby deepening our understanding of the catalytic mechanisms at play.^16,24,26,35^ Notable changes in the Bragg peak intensities were observed in the synchrotron PXRD patterns upon methanol adsorption, as shown in Figure S19 and Table S9. By studying the closest interatomic distances in the Rietveld refined structures, we can gain insights into the interactions between the species of interest. The model revealed the methanol binding site on Cu_1_/Z, as depicted in Figures 5c and S20 and Table S10. The interatomic distances between O_MeOH_ and the Cu site and the closest framework O site were determined to be 3.65(3) Å. On the other hand, two independent methanol binding sites on Cu_3_/Z were revealed, which correspond well with the two Cu_3_ sites present in Cu_3_/Z. Figure 5d shows that an adsorbed methanol is located near Site A, with Cu_I_···O_MeOH_ = 2.40(1) Å and Cu_II_···O_MeOH_ = 2.24(3) Å, indicating coadsorption of the methanol molecule by two Cu moieties. For Site B, the adsorption configuration is similar to that of Site A in terms of interatomic distances (Figure S21 and Table S11). This is in stark contrast to the adsorption of methanol on H-ZSM-5, where the binding of methanol is found solely on the BAS near the framework T6 site (Figure S22). By analyzing the derived Cu···O_MeOH_ distances, the methanol binding is found much closer to the Cu moieties in Cu_3_/Z than in Cu_1_/Z. This suggests that the adsorbed species on Cu_3_/Z is likely methoxy (CH_3_O*) in nature. This observation is consistent with our thermogravimetric analysis-mass spectrometry results (Figure S23), where the methanol binding on Cu_3_/Z is notably stronger than that on Cu_1_/Z. Therefore, facile O–H bond dissociation can occur, suppressing the competing RWGS and MTH reaction pathways. The key differences in the adsorption properties and *in situ* dynamic changes of methanol in the system provide a clear explanation for the observed “metal nuclearity” effect on the basis of our catalysis data set.

Building upon the findings from the adsorption studies, we then performed density functional DFT calculations to validate the observed binding energies and confirm the nature of the intermediates involved in the methanol reforming reaction. We specifically focused on the binding energies of methanol and the methoxy (CH_3_O*) intermediate, as these are critical steps in the methanol reforming reaction.^36^ Figure 5e shows the PDOS of Cux/Z adsorbed with methanol, revealing distinctive p–d orbital overlap behavior between methanol and the Cu sites. Notably, the 2p orbitals of methanol exhibit a strong overlap with the Cu 3d orbital of Cu_3_/Z, indicating strong methanol adsorption on Cu_3_/Z. Consequently, the binding energy of methanol on Cu_3_/Z (−0.65 eV) is much larger than that of Cu_1_/Z (−0.17 eV). Moreover, as shown in Figure 5f, we observed a strong binding of the methoxy intermediate in all three Cu_1_/Z-Cu_3_/Z. This indicates facile dissociation of the O–H bond and further confirms the suppression of the competing reaction pathway, particularly for the RWGS and MTH reactions, as observed in our catalytic results.

## Conclusions

To conclude, we have presented a series of atomically choreographed Cu_*x*_/Z (*x* = 1, 2, and 3) embedded within the microporous channels of ZSM-5 zeolites. The microporosity of the zeolite support offers a crucial role in stabilizing these pseudodiscrete atomic entities, resulting in highly homogeneous local configurations and precise atomic definitions. As illustrated in our model methanol reforming reaction, the substantial difference between the reactive surfaces and geometric structures of the active sites leads to notably different adsorption properties of reaction substrates, rendering sophisticated control of catalytic reactivities and product selectivities. Particularly, we observed tunable and superior catalytic activities, as well as the suppression of the competing RWGS and MTH pathways among the Cu_3_/Z compared to the single-atom Cu_1_/Z analogue. The variations in the adsorption behavior of the reaction substrate and the catalytic data set revealed that the physicochemical properties of solid-atom catalysts could be effectively manipulated through this atomistic engineering approach. Overall, we aimed to gain insights into the effects of structural advantages of the supported Cu_3_ clusters, in terms of the metal nuclearity, on the electronic properties and binding preferences with reaction substrates and their intermediates. Our study successfully demonstrated the potential of atomically choreographed clusters with low nuclearity within the microporous channels of ZSM-5 zeolites, providing valuable insights into their catalytic performance and paving the way for further exploration and optimization of these materials for various applications.

## Supplementary Material



## Notes

Additional experimental details, materials, methods, and additional catalysis and characterization details (PDF)
