# Supplementary material for: Unraveling the Nuclearity Effect of Atomically Choreographed Triatom Cu3 Clusters Supported on Zeolites
Source: J Am Chem Soc. 2025 May 8;147(20):17170–80. doi: 10.1021/jacs.5c02706 (PMC12100646; doi:10.1021/jacs.5c02706)
Supplement: Supplementary file 1 [file ja5c02706_si_001.pdf]

Supplementary information

Unraveling the nuclearity effect of atomically choreographed tri-atom Cu_3 clusters supported on zeolites

Tianxiang Chen[a,b,‡], Yunong Li[a,b,‡], Ping-Luen Ho[c,d,‡], Kwan Chee Leung[c], Jinjie Liu[e], Ching Kit Tommy Wun[a], Zehao Li[f], Chiu Chung Tang[g], Shogo Kawaguchi[h], Tai-Sing Wu[i], Yun-Liang Soo[j], Jun Yin[e,*], Shik Chi Edman Tsang[c,*], Tsz Woon Benedict Lo[a,b,e,k*]

a: State Key Laboratory of Chemical Biology and Drug Discovery, Department of Applied Biology and Chemical Technology, The Hong Kong Polytechnic University, Hung Hom, Hong Kong, China.

b: The Hong Kong Polytechnic University Shenzhen Research Institute, The Hong Kong Polytechnic University, Shenzhen, 518057, China.

c: Inorganic Chemistry Laboratory, Department of Chemistry, University of Oxford, Oxford, OX1 3QR, United Kingdom.

d: Department of Materials, University of Oxford, Oxford, OX1 3PH, United Kingdom.

e: Department of Applied Physics, The Hong Kong Polytechnic University, Hung Hom, Hong Kong, China.

f: School of Chemistry and Chemical Engineering, Anyang Normal University, Anyang, 455000, China.

g: Diamond Light Source Ltd., Harwell Science and Innovation Campus, Didcot, Oxfordshire, Harwell Campus, Oxford, OX11 0DE, United Kingdom.

h: Japan Synchrotron Radiation Research Institute (JASRI), SPring-8, 1-1-1 Kouto, Sayocho, Sayo-gun, Hyogo 679-5198, Japan.

i: National Synchrotron Radiation Research Center, 101 Hsin-Ann Road, Hsinchu 30076, Taiwan.

j: Department of Physics, National Tsing Hua University, Hsinchu 30013, Taiwan.

k: PolyU-Daya Bay Technology and Innovation Research Institute, The Hong Kong Polytechnic University, Huizhou, China

*Corresponding author.

Email addresses: jun.yin@polyu.edu.hk (J. Yin), edman.tsang@chem.ox.ac.uk (S.C.E. Tsang), twblo@polyu.edu.hk (T.W.B. Lo)

Content

Experimental procedures 4
Materials 4
Supplementary Methods...6
Supplementary Figures..12

Figure S1. (**a** – **b**) UV-vis spectra of Cu_1-Z, Cu_2-meIm-Z, and Cu_3-meIm-Z, and (**c** – **d**) the corresponding reduced Cu_1/Z, Cu_2/Z and Cu_3/Z. 12

Figure S2. (**a** – **b**) Thermogravimetric analysis of mononuclear Cu_1/Z, Cu_2-meIm-Z, Cu_3-meIm-Z, and (**c** – **d**) the corresponding hydrogen reduced Cu_1/Z, Cu_2/Z and Cu_3/Z...13

Figure S3. Determination of the change in coordination environment of Cu_3-meIm-Z upon hydrogen reduction by *in-situ* X-ray absorption spectroscopy. Wavelet transform of k^3-weighted EXAFS signal obtained at different temperatures using Morlet wavelet with $\kappa = 10$, $\sigma = 1$. The corresponding Cu K-edge EXAFS (red) and fitting (black and blue), shown in k^3-weighted R-space and k-space, are presented. 15

Figure S4. *In-situ* H_2-EXAFS of Cu_2-meIm-Z under a hydrogen reduction atmosphere. Fourier transform of EXAFS spectra in the R-space from 303 K to 623 K........................16

Figure S5. Determination of the change in coordination environment of Cu_2-meIm-Z upon hydrogen reduction by *in-situ* X-ray absorption spectroscopy. Wavelet transform of k^3-weighted EXAFS signal obtained at different temperatures using Morlet wavelet with $\kappa = 10$, $\sigma = 1$. The corresponding Cu K-edge EXAFS (red) and fitting (black and blue), shown in k^3-weighted R-space and k-space, are presented. 18

Figure S6. XPS analyses of (**a** − **c**) Cu_1-Z, Cu_2-meIm-Z, and Cu_3-meIm-Z, and (**d** − **f**) the reduced Cu_1/Z, Cu_2/Z, and Cu_3/Z. 19

Figure S7. H_2-TPR profiles of Cu_1/Z, Cu_2/Z, Cu_3/Z (from their corresponding meIm-mediated precursors) and $Cu(OAc)_2$. 21

Figure S8. H_2-TG and the corresponding differential TG curves of Cu_3-meIm-Z. 22

Figure S9. copper content and the Cu/Al molar ratio of Cu_x/Z (x = 1, 2, and 3), and Cu_1/Z[2] (‘Cu_1/Z[2]’: ion-exchange with Cu ions for two times without ligand molecules). 23

Figure S10. (**a**) Synchrotron PXRD data of Cu_x/Z, and (**b** − **d**) detailed comparison of the synchrotron PXRD patterns of the samples. 25

Figure S11. Fourier difference map derived by charge flipping (by TOPAS-v7.0) that determines the locations of the heavier atoms along the (**a**) [010], and (**b**) [100] zone axes. 26

Figure S12. High-loss EELS fine structure of copper intercalated into the Cu_3/Z. 27

Figure S13. The projected density of states (PDOS) of the reduced (**a**) Cu_1/Z, (**b**) Cu_2/Z, (**c**) Cu_3/Z, and (**d** − **f**) the corresponding crystal structures. (blue = Si, red =O, and orange = Cu). 28

Figure S14. Photograph of the batch reactor (BE100 reactor, Shanghai LABE Instrument Co,. Ltd.). 29

Figure S15. Catalytic performance evaluation of Cu_3/Z at different reaction temperatures, with a methanol-to-water ratio of 1:1. 30

Figure S16. Catalytic performance evaluation of Cu_3/Z at different methanol-to-water ratios. Reaction temperature = 523 K. 31

Figure S17. (**a**) Synchrotron PXRD data of Cu_3/Z and Cu_3/Z pre-adsorbed with methanol (Cu_3/Z+MeOH) (E = 18 keV, λ = 0.688657 (2) Å), and (**b** − **d**) comparison of the diffraction patterns of the samples in detail. 32

Figure S18. Synchrotron PXRD patterns and the Rietveld refinement profile of Cu_1/Z+MeOH.33
Figure S19. Synchrotron PXRD patterns and the Rietveld refinement profile of Cu_3/Z (Site B) pre-adsorbed with methanol. 34
Figure S20. Synchrotron PXRD patterns and the Rietveld refinement profile of pristine ZSM-5 pre-adsorbed with methanol. 35
Figure S21. TG-MS curves of Cu_1/Z and Cu_3/Z with pre-adsorbed methanol. 36
Supplementary Tables 37
Table S1. Coordination environments derived from *in-situ* X-ray absorption spectroscopy; quantitative fitting parameters of Cu_3-meIm-Z. CN is the average coordination number around the central atoms. R and σ^2 are the average bond distance and the Debye-Waller factor, respectively. The ΔE_0 values of two Cu–X shells are constrained to share the same value in the fitting models. 37
Table S2. Coordination environments derived from *in-situ* X-ray absorption spectroscopy; quantitative fitting parameters of Cu_2-meIm-Z. CN is the average coordination number around the central atoms. R and σ^2 are the average bond distance and the Debye-Waller factor, respectively. The ΔE_0 values of two Cu–X shells are constrained to share the same value in the fitting models. 38
Table S3. Elemental analysis of Cu_x/Z by ICP-OES. 40
Table S4. Crystallographic parameters of Cu_x/Z from the Rietveld refinement of synchrotron PXRD data. 41
Table S5. Atomic parameters of Cu_1/Z from the Rietveld refinement of synchrotron PXRD data. 42
Table S6. Atomic parameters of Cu_2/Z from the Rietveld refinement of synchrotron PXRD data. 44
Table S7. Atomic parameters of Cu_3/Z from the Rietveld refinement of synchrotron PXRD data. 46
Table S8. Crystallographic parameters of Cu_1/Z and Cu_3/Z pre-adsorbed with methanol. 48
Table S9. Atomic parameters of Cu_1/Z+MeOH from the Rietveld refinement of synchrotron PXRD data. The sample was dried at 100 °C overnight to remove physisorbed methanol species. 49
Table S10. Atomic parameters of Cu_3/Z+MeOH from the Rietveld refinement of synchrotron PXRD data. The sample was dried at 100 °C overnight to remove physisorbed methanol species. 51
Supplementary References 53

Experimental procedures

Materials

H-ZSM-5 zeolites. Commercial H-ZSM-5 zeolites (SiO_2:Al_2O_3 = 46, chemical formula $H_nAl_nSi_{96-n}O_{192}·18H_2O$) were purchased from Nankai University Catalyst Co., Ltd. Typical characterization results are displayed on their product website: [http://www.nkcatalyst.com/index.php/en/arc/show/id/49.html]. The H-ZSM-5 sample possesses high crystallinity (≥ 95%), a surface area of *ca*. 340 $m^2·g^{-1}$, and a pore size of 5.3 Å × 5.6 Å.

Sources of metal ion precursor. All the metal ion sources ($Co(NO_3)_2·6H_2O$, $Ni(NO_3)_2·6H_2O$, $Cu(NO_3)_2·2.5H_2O$ and $Zn(NO_3)_2·6H_2O$) and 2-methylimidazole (meIm) were purchased from Sigma Aldrich and used as received without further purification.

Synthesis of tri-atom catalysts (TACs). Take the homometallic copper-based catalysts as an example:

1. *Ligand-mediation*

Cu_1-Z: Cu_1-Z was synthesized *via* a conventional ion exchange process. 2.0 g H-ZSM-5 (SiO_2:Al_2O_3 = 46) was suspended in 0.2 M $Cu(NO_3)_2$ solution (60 mL) for 20 h at 353 K. Then the solid 'Cu_1-Z' sample was washed and collected after overnight drying at 343 K.

Cu_2-meIm-Z. 2.0 g Cu_1-Z sample was suspended in 60 ml deionized water and mixed with 0.052 g meIm. The suspension was stirred for 20 h at 353 K. The solid Cu_1-meIm-Z sample was washed extensively and collected after overnight drying at 343 K. It was then suspended in 60 mL 0.2 M $Cu(NO_3)_2$ solution. The suspension was allowed to be stirred for 20 h at 353 K. The resultant 'Cu_2-meIm-Z' sample was washed extensively and collected after overnight drying at 343 K.

Cu_3-meIm-Z: 2.0 g Cu_2-meIm-Z was dispersed in 60 mL DI water and mixed with 0.052 g meIm. The suspension was allowed to be stirred for 20 h at 353 K. It was then washed extensively and collected after overnight drying at 343 K. Extra attention should be drawn to this step. More washing steps using methanol as a solvent are needed to fully wash the samples. It was then suspended in 60 mL 0.2 M $Cu(NO_3)_2$ solution. The suspension was allowed to be stirred for 20 h at 353 K. The solid Cu_3-meIm-Z sample was washed extensively and collected after overnight drying at 343 K.

2. *Sample reduction*

Cu_x/Z (x =1, 2 and 3): 1.0 g Cu_1-Z, Cu_2-meIm/Z, or Cu_3-meIm/Z was placed in a quartz boat and centered in a quartz tube furnace (BTF-1200C, Anhui BEQ Equipment Technology Co., Ltd.). The reduction treatment was conducted at 573 K within a temperature ramping rate of 5 K min^{-1} for 1 h under the atmosphere of 10% H_2/N_2 with a gas flow rate of 300 mL min^{-1}. The

reduced sample was collected and denoted as ‘Cu_1/Z’, ‘Cu_2/Z’, or ‘Cu_3/Z’, and stored in a vacuum oven for further usage.

Supplementary Methods

Inductively Coupled Plasma Optical Emission Spectroscopy (ICP-OES). The chemical composition of samples was determined using a 5900 ICP-OES from Agilent Technologies. For the ICP-OES spectroscopy analysis, zeolite powder of 0.5 mg was first dissolved in the mixture of HF solution (2 mL, 39%, Merck) and aqua regia for at least 12 h under stirring before dilution and filtration using 0.45-µm microfilter. Boric acid was added to minimize fluoride interferences during measurement.

Synchrotron X-ray Powder Diffraction (PXRD) and Rietveld Refinement. Synchrotron PXRD measurements for structure determination were collected on BL02B2 at SPring-8 [1] Japan and Beamline I11 at Diamond Light Source, United Kingdom. The wavelength and the 2θ zero-point were calibrated using a diffraction pattern obtained from the CeO_2 standard (NIST SRM674b) and Si powder standard (NIST SRM640c), respectively. High-throughput synchrotron PXRD data were obtained from the zeolite samples (loaded in 0.5-mm borosilicate capillaries) using the MYTHEN detectors with 70 ° aperture. The patterns were collected in the 2θ range 2-70 ° with 0.006 ° data binning. Each synchrotron PXRD data was collected for 10 min to produce a good quality pattern with a high signal-to-noise ratio. The synchrotron PXRD data was analyzed using the Rietveld refinement method available in TOPAS-v7.0 software. [2]

The lattice parameters were obtained using the analytical software, and the background curve was fitted by a Chebyshev polynomial with an average of 20 coefficients. The Thompson-Cox-Hastings (pseudo-Voigt) function was applied to describe the diffraction peaks. [3] The scale factor and lattice parameters were allowed to vary for all the histograms. Each data histogram's final refined structural parameters were carried out using the Rietveld method with the fractional coordinates (*x, y, z*) and isotropic displacement factors (Beq) for all atoms.

1. Framework atoms

With the introduction of metal ions and organic molecules, the positions of the framework atoms may change slightly. Therefore, before the refinement of the entire structure with guest metal ions and organic linker molecules, the framework atoms were first refined to avoid a miscalculation of the structure that reaches the global minimum of the refinement by changing the entire framework. The fractional coordinates and the Beq of the framework atoms (Si and O) were fixed as performed in our previous work [4–6], and all other parameters were refined over the 2θ range of 15-55 °.

2. Fourier analysis

Over the 2θ range of 3-55 °, the Fourier analysis was used to identify the positions with the highest remaining electron density in the framework once the positions of the framework atoms have been refined.

3. Inclusion of guest molecules

After identifying the crystallographic location of the metal sites, the site occupancy factors (SOFs) of the metal sites were constrained based on the elemental results from the elemental analysis. The SOFs of the linkers were restrained based on the SOFs of the metal sites. The fractional atomic coordinates and the SOFs of linker molecules were allowed to be refined freely. Finally, the relevant atomic parameters were relaxed and refined by simulated annealing for an hour to ensure the global minimum was reached. The lowest R_{wp} and gof (goodness-of-fit) values indicate the global minimum.

X-ray Absorption Spectroscopy. The X-ray absorption spectroscopy data were collected on Beamline 07A at Taiwan Photon Source at the National Synchrotron Radiation Research Center, Taiwan. A Si(111) double crystal monochromator was used to scan the photon energy. Artemis and Athena software were used for data treatment and analysis. [7] The detailed fitting parameters are summarized in the caption of the EXAFS fittings. The Hamma software was used for the wavelet transform. [8]

In-situ X-ray Absorption Spectroscopy. The *in-situ* X-ray absorption spectroscopy was collected on Beamline 07A at Taiwan Photon Source at the National Synchrotron Radiation Research Center, Taiwan. The samples were reduced under the atmosphere of 5 % H_2/Ar with the temperature increased by 3 K min^{-1} from room temperature to 623 K. A Si(111) double crystal monochromator was used to scan the photon energy. Artemis and Athena software were used for data treatment and analysis. [7] The detailed fitting parameters are summarized in the caption of the EXAFS fittings. The Hamma software was used for the wavelet transform. [8]

Scanning Transmission Electron Microscopy (STEM). The STEM images were taken using an aberration-corrected transmission electron microscope of JEM-ARM 300F from JEOL. The microscope worked at the high voltage of 300 kV, with the collected semi-angles for the annular detector in the STEM mode ranging from 47 mrad to 221 mrad. The probe array sizes of 1024 × 1024 and a dwell time of 0.3 μs per pixel were used during the experiments. The probe current was set at a range of 12-15 pA. Given that the beam-sensitive zeolitic framework can be easily damaged and forms amorphous contamination when the incident electron beams interact with the sample, the adjustment of probe current to approach low dose and scanning the target specimen from the edge were used during imaging.

Ultraviolet-Visible-Near-Infrared Diffuse Reflectance Spectroscopy (UV-vis-NIR DRS). The UV-vis-NIR DRS measurements were carried out with the obtained samples using a Perkin-Elmer Lambda 1050-UV-Vis-NIR spectrophotometer with an Integrating Sphere-150 mm UV-vis-NIR (InGaAs) Module. The equipment was calibrated using the Spectralon standard, and the reflectance was measured in the 200 − 2500 nm range at an interval of 2 nm.

X-ray Photoelectron Spectroscopy (XPS). The XPS measurements were performed using a Thermo Scientific Nexsa spectrometer (12 kV cathode-biased Al K_α, hv = 1486.6 eV) with a spot size of 400 μm at the base pressure < 5 × 10^{-10} mbar. An argon ion gun was used to etch the samples with an etching rate of 0.5 nm s^{-1}. The XPS spectra were obtained at approximately

20 nm depth intervals. The raw data were corrected for substrate charging with the binding energy of the C 1*s* peak at 284.8 eV.

Hydrogen Temperature-Programmed Reduction (H_2-TPR). The H_2-TPR measurements were performed using Micromeritics AutoChem II 2920 H_2-TPR. The measurement steps are as follows. 100 mg of the sample was weighed and placed in a U-shaped quartz tube. The pre-treatment was carried out at a ramping rate of 10 K min^{-1} from room temperature to 473 K in a flow of He (50 ml min^{-1}). This process necessitated a duration of 2 h, leading to the desiccation of the samples. Then the sample was cooled to 323 K, and a 10% H_2/Ar mixture was introduced at the flow rate of 50 mL min^{-1} for 0.5 h until the baseline stabilized. The TPR measurement was carried out at a ramping rate of 10 K min^{-1} from 323 K to 1073 K in a flow of 10 % H2/Ar mixture (50 ml min^{-1}). A thermal conductivity detector (TCD) was employed to detect the released gases.

Thermogravimetric Analysis (TGA). The TGA measurements were performed using a Thermogravimetric analyzer/Differential Scanning Calorimeter (Mettler Toledo TGA/DSC3+). Around 10 mg zeolitic sample was weighed and placed in a corundum crucible; the sample was heated from room temperature to 1173 K within a ramping rate of 10 K min^{-1} in a flow of N_2 (40 mL min^{-1}).

The TGA measurements were performed using the same equipment setup for the methanol pre-adsorbed on zeolite samples. The apparent activation energy for the desorption of methanol is estimated according to the following modified Arrhenius equation (**Supplementary Text 1**): [9]

$$2lnT_m - ln\beta = \frac{E_{des}}{RT_m} + ln\frac{E_{des}}{AR}$$

where T_m is the peak desorption temperature at a specific β, β is the temperature ramping rate, E_{des} is the desorption energy and A is a numerical constant. Plotting ($2\ln T_m - \ln\beta$) versus $1/T_m$ for a series of β values allows the estimation of E_{des}.

Hydrogen Thermogravimetric (H_2-TG) Analysis. The H_2-TG analyses were performed using Mettler Toledo TGA/DSC 3+. Around 10 mg zeolitic sample was weighed and placed in a corundum crucible, and the sample was heated from room temperature to 723 K within a ramping rate of 10 K min^{-1} in a flow of 5 % H_2/Ar mixture (50 mL min^{-1}).

Thermogravimetric Analysis-Mass Spectroscopy (TG-MS). The TG-MS measurements were performed using Rigaku thermo plus EV2/thermo mass photo. The sample was heated from room temperature to 823 K within a ramping rate of 10 K min^{-1} in a flow of N_2 (40 mL min^{-1}). The desorbed gas was analyzed by the embedded mass spectrometer.

***In-situ* Diffuse Reflectance Infrared Fourier Transform Spectroscopy (*in-situ* DRIFTS).** The *in-situ* DRIFTS measurements were performed using Nicolet IS50 with an MCT-A detector (data mode: Kubelka-Munk). The scan times were set 128 times (changed to 64 times

during the heating process) with a data resolution of 8 cm^{-1}, and the measurement range was set at 4000 – 650 cm^{-1}.

1. background collection

The samples were kept in a flow of N_2 (60 ml min^{-1}) with a heating rate of 10 K min^{-1} in the range of room temperature to 673 K. This process was maintained for 2 h for dehydration. The background and a reference IR were collected after a 5-minute heat preservation at specific temperatures (673, 648, 623, 598, 573, 548, 523, 498, 473, 448, 423, 398, 373, 348 K and room temperature).

2. the in-situ process

The atmosphere was built by N_2 purging methanol solution at a flow rate of 30 mL min^{-1}, and the data was collected at the same temperatures as the background. The measurement steps are as follows. The dehydration was carried out in a flow of N_2 (60 ml min^{-1}), with a ramping rate of 10 K min^{-1} from room temperature to 673 K and kept for 2 h. The temperature was then dropped to a specific temperature (673, 648, 623, 598, 573, 548, 523, 498, 473, 448, 423, 398, 373, 348 K and room temperature) and preserved the heat for 5 mins, then the background and a reference IR were collected. The dehydrated sample with the cell was kept at 348 K, adsorbing methanol vapor (externally heated by constant temperature water bath) carried by N_2 for 1 h. After the adsorption of inert gas, purge the sample for 10 minutes and then collect the FT-IR data. Then the FT-IR measurements were carried out in a flow of N_2 (60 ml min^{-1}) at a ramping rate of 10 K min^{-1} from room temperature to 673 K. During the heating process, the FT-IR data was collected after a 5-minute heat preservation period at specific temperatures (373, 398, 423, 448, 473, 498, 523, 548, 573, 598, 623, 648, and 673 K). Cooled the sample down to room temperature and collected the FT-IR data.

Density Functional Theory (DFT) Calculations. We performed DFT calculations using the projector-augmented wave method implemented in the Vienna Ab initio Simulation Package (VASP). The generalized gradient approximation (GGA) with the Perdew-Burke-Ernzerhof (PBE) exchange-correlation functional was employed. A uniform 2×2×2 *k*-mesh grid in the Brillouin zone was used to optimize the crystal structures of TACs/Z, and the kinetic energy cutoff for the wave functions was set at 450 eV before and after CH_3OH (or CH_3O^*) adsorption. The atomic positions of all crystal structures were relaxed until the forces on each atom were less than 0.015 eV/Å. The same DFT setting was used to obtain the projected density of states (PDOS) for all TACs/Z crystal structures.

The binding energy for methanol and CH_3O^* adsorption was calculated as

$E(\text{binding}) = E(\text{TACs/Z} + CH_3OH) - E(\text{TACs}) - E(CH_3OH)$

$E(\text{binding}) = E(\text{TACs/Z} + CH_3O^*) - E(\text{TACs}) - E(CH_3O^*)$

where $E(\text{TACs/Z} + CH_3OH)$ and $E(\text{TACs/Z} + CH_3O^*)$ are the total energies of the TACs/Z with CH_3OH and CH_3O^* adsorption, respectively; E(TACs/Z) are the energies of TACs/Z; $E(CH_3OH)$ and $E(CH_3O^*)$ are the energies of CH_3OH and CH_3O^*, respectively.

Evaluation of catalytic performance

Experimental Procedures

$$CH_3OH \xrightarrow[\text{MeOH/H}_2\text{O, N}_2 \\ \text{10 bar, 573 K, 24 h}]{\text{Cu}_x\text{/Z (x = 1, 2, and 3)}} H_2 + CO + CO_2 + C_2\text{~}C_4\text{+}$$

The methanol reforming reaction was carried out using a batch reactor (**Figure S15**, BE100 reactor, Shanghai LABE Instrument Co., Ltd.). The pre-treated catalysts of 125 mg were placed into a quartz lining containing 5 mL of solution (the reaction temperature and volume ratio of H_2O/CH_3OH varies from 1:1 to 1:8.5, see **Figures S16 – S17**). Before the reaction, the reactor was purged with 99.999% N_2 by filling and deflating it three times, reaching a pressure of 10 bar. The reaction was conducted at temperatures of 473, 523, and 573 K, with a reaction duration of 24 h. Gas products were quantified by Gas chromatography (GC) spectroscopy (TDX-01 column, GC-MS 2060, Shanghai Ruimin Instrument Co., Ltd.) equipped with a flame ionization detector (FID) and a thermal conductivity detector (TCD).

Calculation of the reaction pathway preferences

$$(CH_x)_m \xleftarrow{\textbf{MTH}} CH_3OH \xrightarrow{\textbf{Core reforming}} CO_2 + H_2 \xrightarrow{\textbf{RWGS}} CO$$

The methanol reforming reaction serves as an effective model to explore reactivity descriptors due to the presence of multiple competing reaction pathways, including core reforming (yielding CO_2 and H_2), subsequent reverse water-gas shift (RWGS, yielding CO and H_2O), and methanol-to-hydrocarbons (MTH). In the RWGS pathway, the CO_2 and H_2 obtained from the core reforming will be consumed and converted to CO. Calculating the pathway preference for core reforming is based on the ratio of the residual CO_2 (yield of the CO_2 detected from the GC) to the sum of the carbon products from the three reaction pathways. The equations are shown below:

$$Preference_{core\ reforming}\ (\%) = \frac{Yield\ of\ (CO_2)}{Yield\ of\ (CO_2 + CO + (CH_x)_m)} \times 100\%$$

$$Preference_{RWGS}\ (\%) = \frac{Yield\ of\ CO}{Yield\ of\ (CO_2 + CO + (CH_x)_m)} \times 100\%$$

$$Preferences_{MTH}\ (\%) = \frac{Yield\ of\ ((CH_x)_m)}{Yield\ of\ (CO_2 + CO + (CH_x)_m)} \times 100\%$$

The preferences of the pathway were present in the form of a pie chart (see **Figure 3** and **Figure 4** in the manuscript), where the area of the sector is the preference of each pathway.

Poisoning experiment

The poisoning experiment was conducted on the reduced sample Cu_3/Z. 100 mg Cu_3/Z was suspended in 0.1 M NaCl solution (3 mL) for 24 h at 353 K. Then the solid 'Cu_3/Na-Z' (where Na^+ replaces H^+) sample was washed and collected after overnight drying at 343 K. The experiments were conducted with these two catalysts under identical conditions (with the reaction temperature at 573 K).

Supplementary Figures

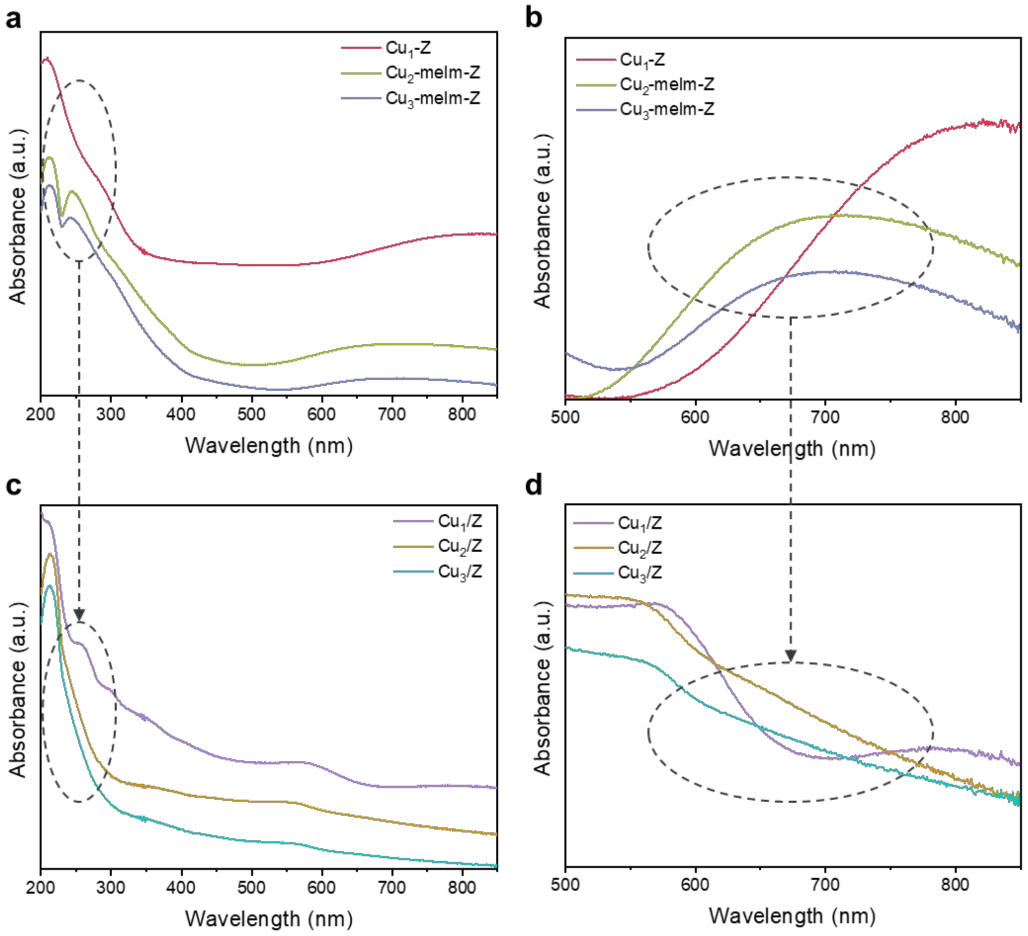

Figure S1. (**a – b**) UV-vis spectra of Cu_1-Z, Cu_2-meIm-Z, and Cu_3-meIm-Z, and (**c – d**) the corresponding reduced Cu_1/Z, Cu_2/Z and Cu_3/Z.

To determine the change in the oxidation state of Cu sites (*d-d* transition, 500 − 800 nm) of Cu(II) and the ligand-metal charge transfer (meIm-Cu interaction, 200 − 300 nm), we analyzed the UV-vis spectra of Cu_1-Z, Cu_2-meIm-Z, Cu_3-meIm-Z, and the corresponding reduced samples Cu_x/Z (**Figure S1**). After hydrogen reduction at 573 K, the absorption band around 270 nm diminished, suggesting the removal of linker molecules. Another absorption band located around 800 nm is also absent, indicating the Cu(II) reduction behavior.

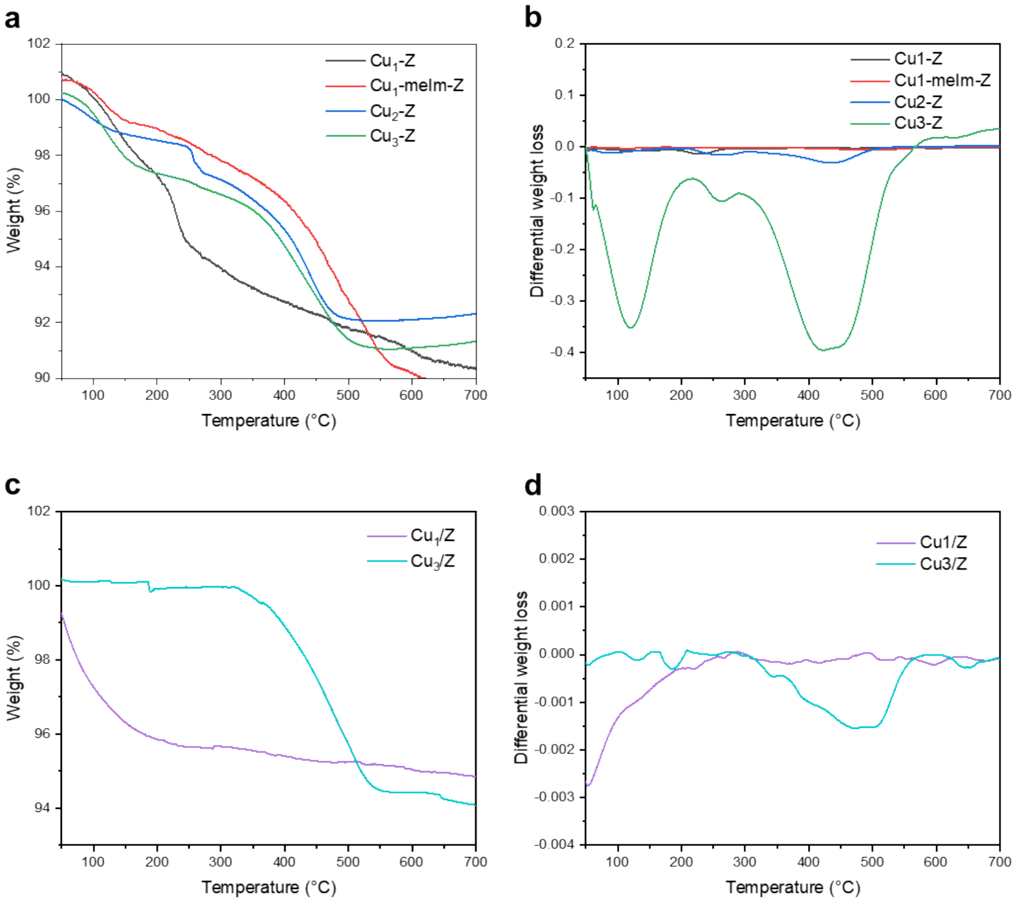

Figure S2. (**a – b**) Thermogravimetric analysis of mononuclear Cu_1/Z, Cu_2-meIm-Z, Cu_3-meIm-Z, and (**c – d**) the corresponding hydrogen reduced Cu_1/Z, Cu_2/Z and Cu_3/Z.

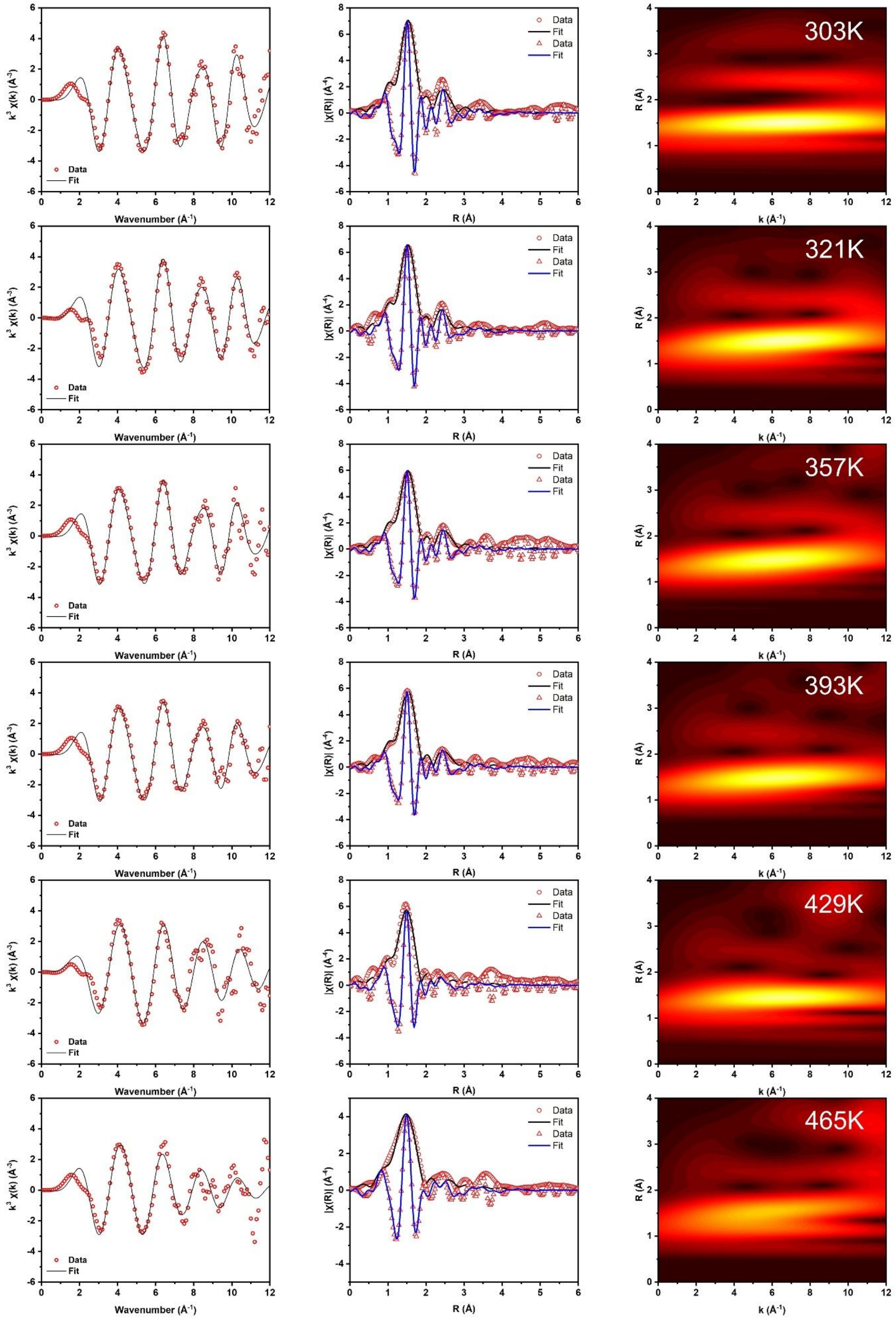
303K
321K
357K
393K
429K
465K
Data
Fit
Wavenumber (Å-1)
k3 χ(k) (Å-3)
R (Å)
|χ(R)| (Å-4)
k (Å-1)

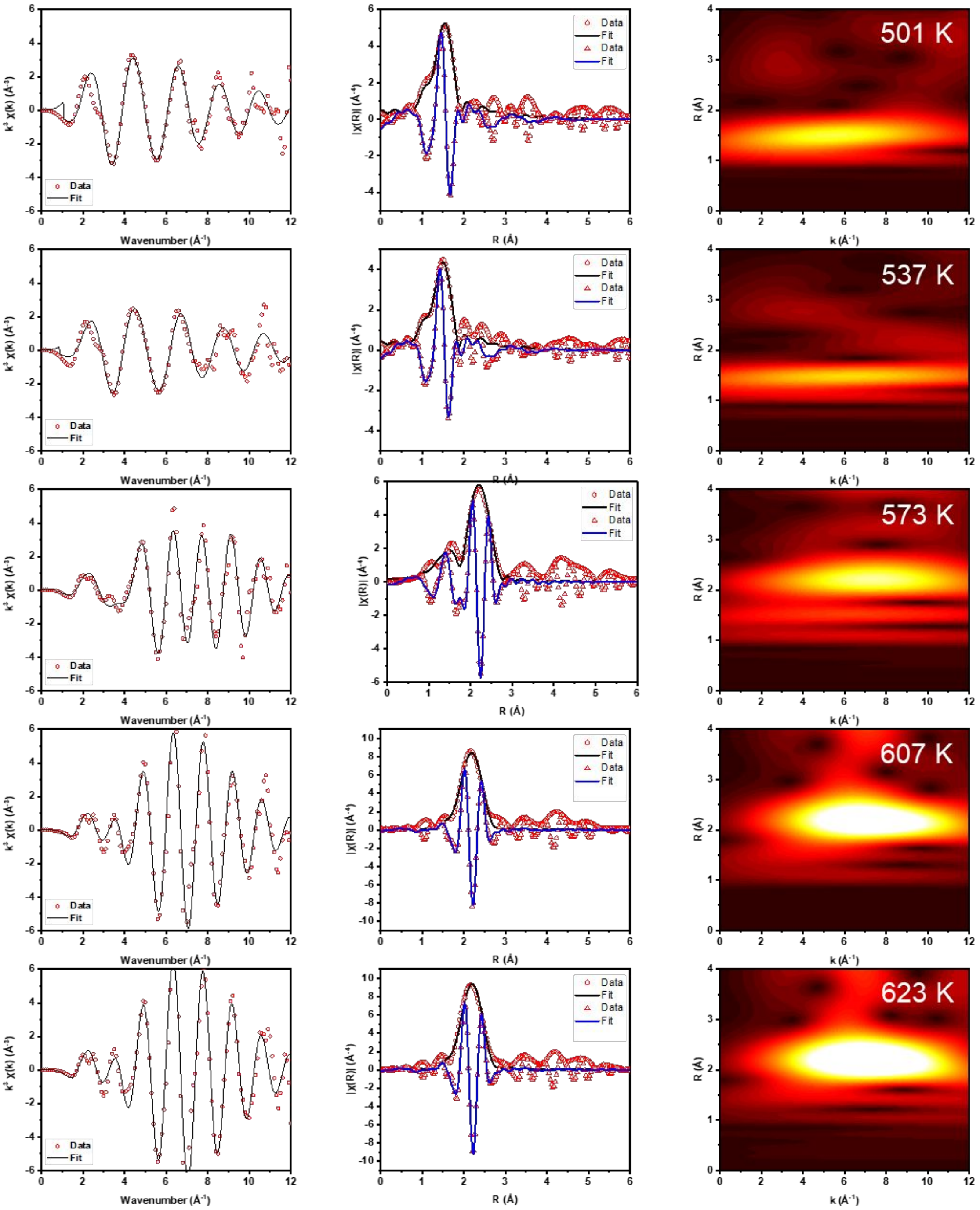

Figure S3. Determination of the change in coordination environment of Cu_3-meIm-Z upon hydrogen reduction by *in-situ* X-ray absorption spectroscopy. Wavelet transform of k^3-weighted EXAFS signal obtained at different temperatures using Morlet wavelet with $\kappa = 10$, $\sigma = 1$. The corresponding Cu K-edge EXAFS (red) and fitting (black and blue), shown in k^3-weighted R-space and k-space, are presented.

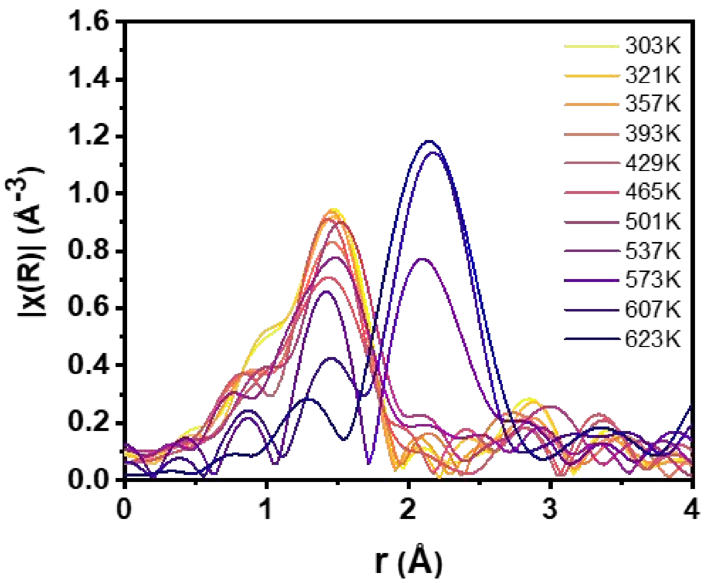

Figure S4. *In-situ* H_2-EXAFS of Cu_2-meIm-Z under a hydrogen reduction atmosphere. Fourier transform of EXAFS spectra in the R-space from 303 K to 623 K.

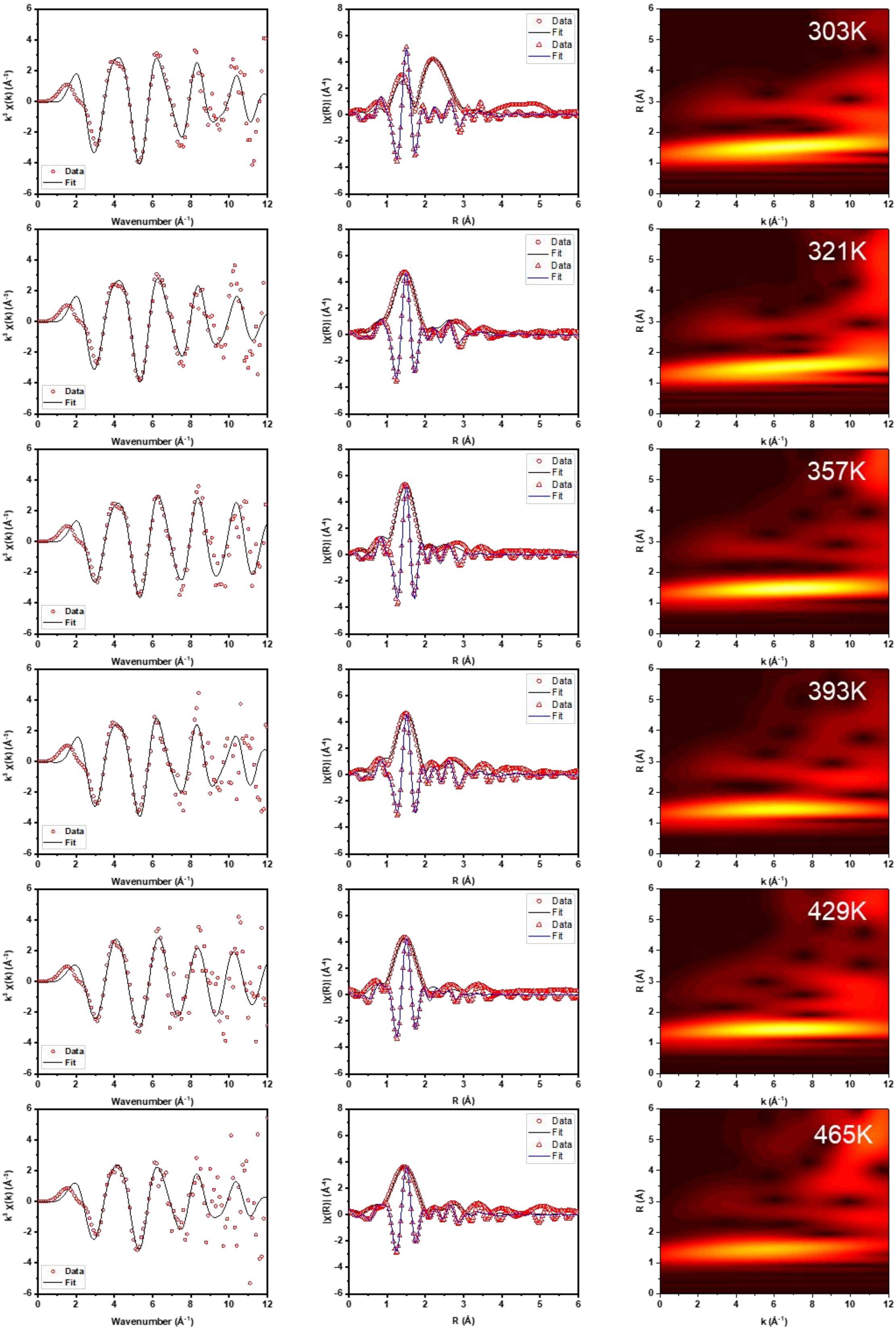

303K
321K
357K
393K
429K
465K
Data
Fit
Wavenumber (Å⁻¹)
R (Å)
k (Å⁻¹)

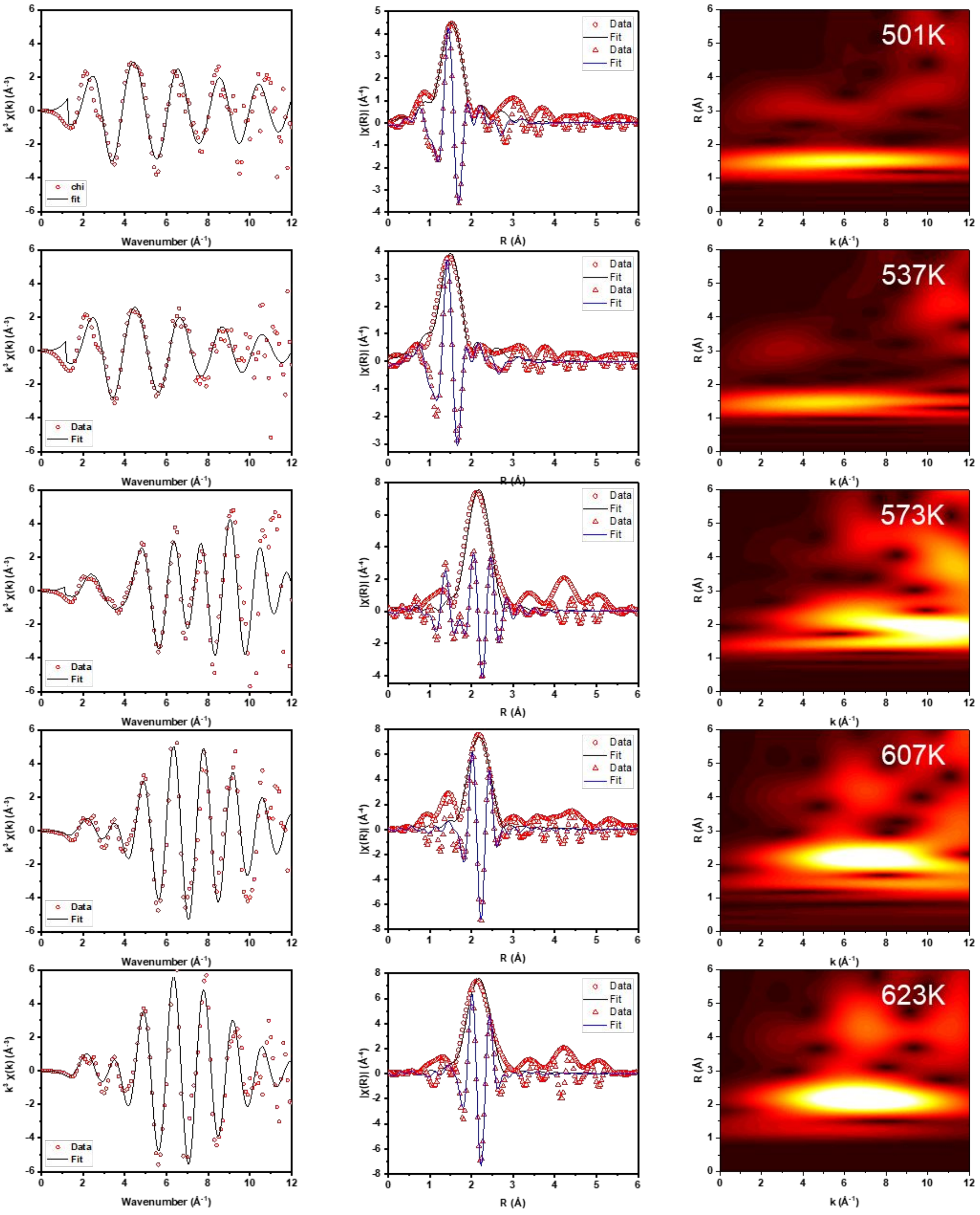

Figure S5. Determination of the change in coordination environment of Cu_2-meIm-Z upon hydrogen reduction by *in-situ* X-ray absorption spectroscopy. Wavelet transform of k^3-weighted EXAFS signal obtained at different temperatures using Morlet wavelet with κ = 10, σ = 1. The corresponding Cu K-edge EXAFS (red) and fitting (black and blue), shown in k^3-weighted R-space and k-space, are presented.

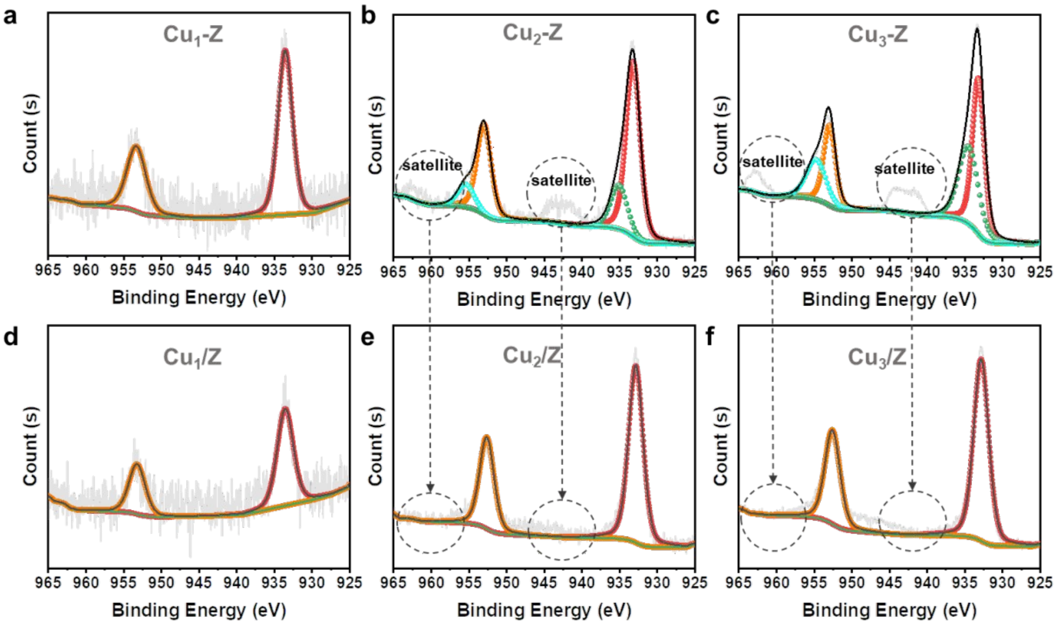

Figure S6. XPS analyses of (**a − c**) Cu_1-Z, Cu_2-meIm-Z, and Cu_3-meIm-Z, and (**d − f**) the reduced Cu_1/Z, Cu_2/Z, and Cu_3/Z.

As shown in **Figure S6**, we observed pairs of Cu $2p_{1/2,3/2}$ doublets and two satellite peaks in the XPS of Cu_2-meIm-Z (Cu $2p_{3/2}$ = 933.06 eV and 935.57 eV) and Cu_3-meIm-Z (Cu $2p_{3/2}$ = 933.11 eV and 934.52 eV). The presence of satellite peaks, along with the two fitting peaks, provides strong evidence for the oxidation states of the Cu species in the Cu_2-meIm-Z and Cu_3-meIm-Z clusters. The satellite peaks indicate the presence of Cu(II) species, suggesting that some of the copper atoms within the clusters have an oxidation state of +2. Additionally, the two fitting peaks further support this conclusion, indicating the presence of Cu(I) species with an oxidation state of +1. Therefore, the combined evidence from the satellite peaks and fitting peaks confirms that the Cu species in the Cu_2-meIm-Z and Cu_3-meIm-Z clusters exist in both +1 and +2 oxidation states. The co-existence of the Cu(I) and Cu(II) peaks suggests that the coordination environment of the two meIm-mediated copper atoms may be different (**Figure S6b − c**), Cu^+ is coordinated with the pyridine-like nitrogen (sp^2-hybridized C=N, electron-withdrawing) of imidazole, while Cu^{2+} is coordinated with the pyrrole-like nitrogen (conjugated, electron-donating). Only one pair of the doublets was observed in Cu_2/Z (Cu $2p_{3/2}$ 932.86 eV, Cu $2p_{1/2}$ = 952.64 eV) and Cu_3/Z (Cu $2p_{3/2}$ = 932.75 eV, Cu $2p_{3/2}$ = 952.53 eV), respectively. The absence of satellite peaks and the lower binding energy (around 932.80 eV) indicates that the reduction process effectively removed the coordinated linker molecules from the Cu sites and the reduction of Cu^{2+} species.

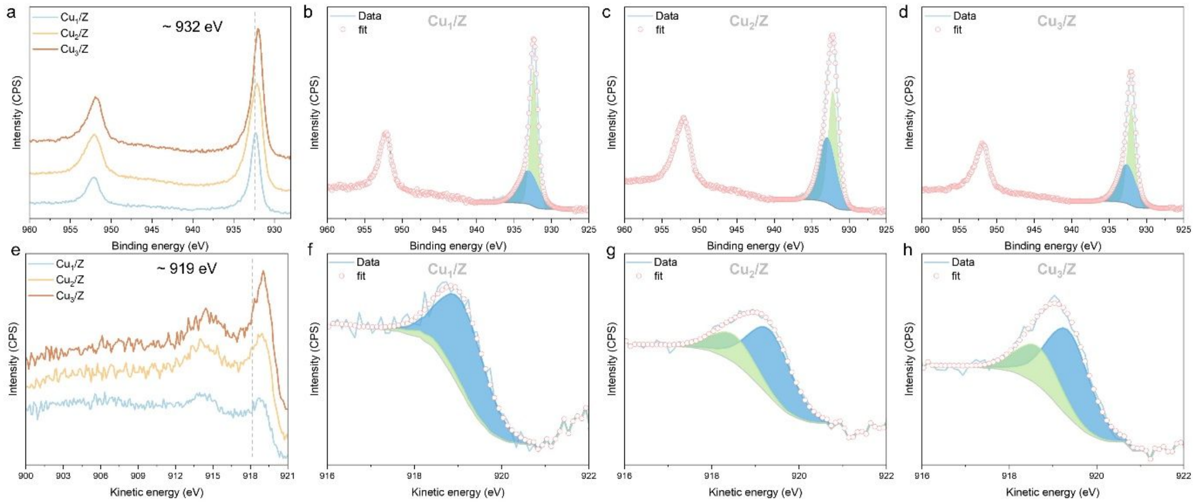

Figure S7. (**a − d**) Cu 2p XPS spectra, and (**e − h**) Cu LMM Auger electron spectra of reduced Cu_x/Z.

All three samples exhibited a peak around 932 eV (**Figure S7a − d**) and no satellite peak was observed, indicating the reduction of Cu^{2+}. A characteristic peak located around 919 eV was observed in the Cu LMM spectra of Cu_1/Z, Cu_2/Z, and Cu_3/Z, further supporting the reduced copper species (metallic Cu^0 or Cu^{1+}, **Figure S7e − h**). The asymmetric $2p_{3/2}$ peak of Cu_x/Z (x = 1, 2, and 3) and the Cu LMM auger spectra were analyzed (**Figure S7b – d, and f − h**), and the distinct Cu(0):Cu(I) ratios have been summarized in **Table S1**. The Cu(0) and Cu(I) concentrations are 58.7% and 41.3% of Cu_1/Z, 48.1% and 51.9% of Cu_2/Z, and 50.5% and 49.5% of Cu_3/Z based on the Cu $2p_{3/2}$ spectra fitted result. The Cu LMM Auger electron spectra fitted result demonstrated a Cu(0) ratio and Cu(I) ratio of 90.2% and 9.8% of Cu_1/Z, 70.8% and 29.2% of Cu_2/Z, and 69.1% and 30.9% of Cu_3/Z. Given the Cu LMM Auger electron spectroscopic result, it can be further deduced that the overall charge state of Cu_3/Z is close to +1.

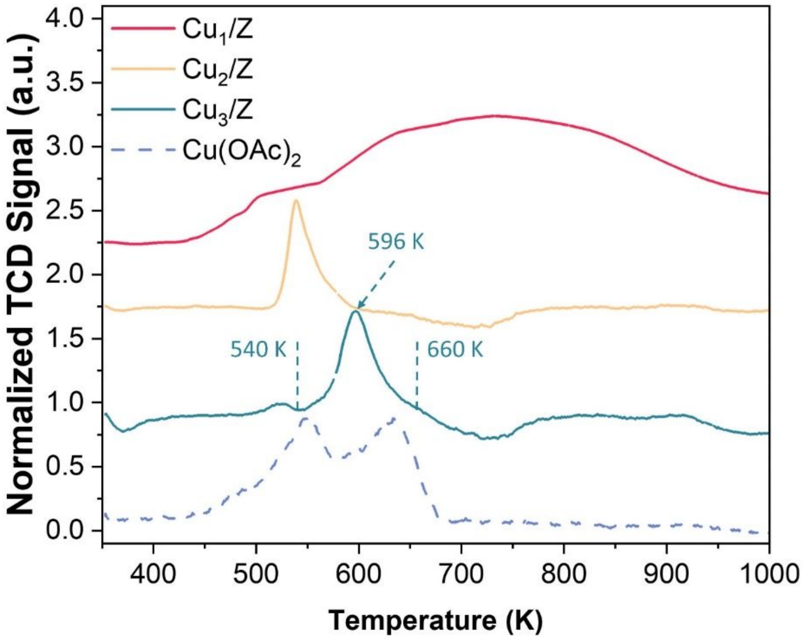

Figure S8. H_2-TPR profiles of Cu_1/Z, Cu_2/Z, Cu_3/Z (from their corresponding meIm-mediated precursors) and $Cu(OAc)_2$.

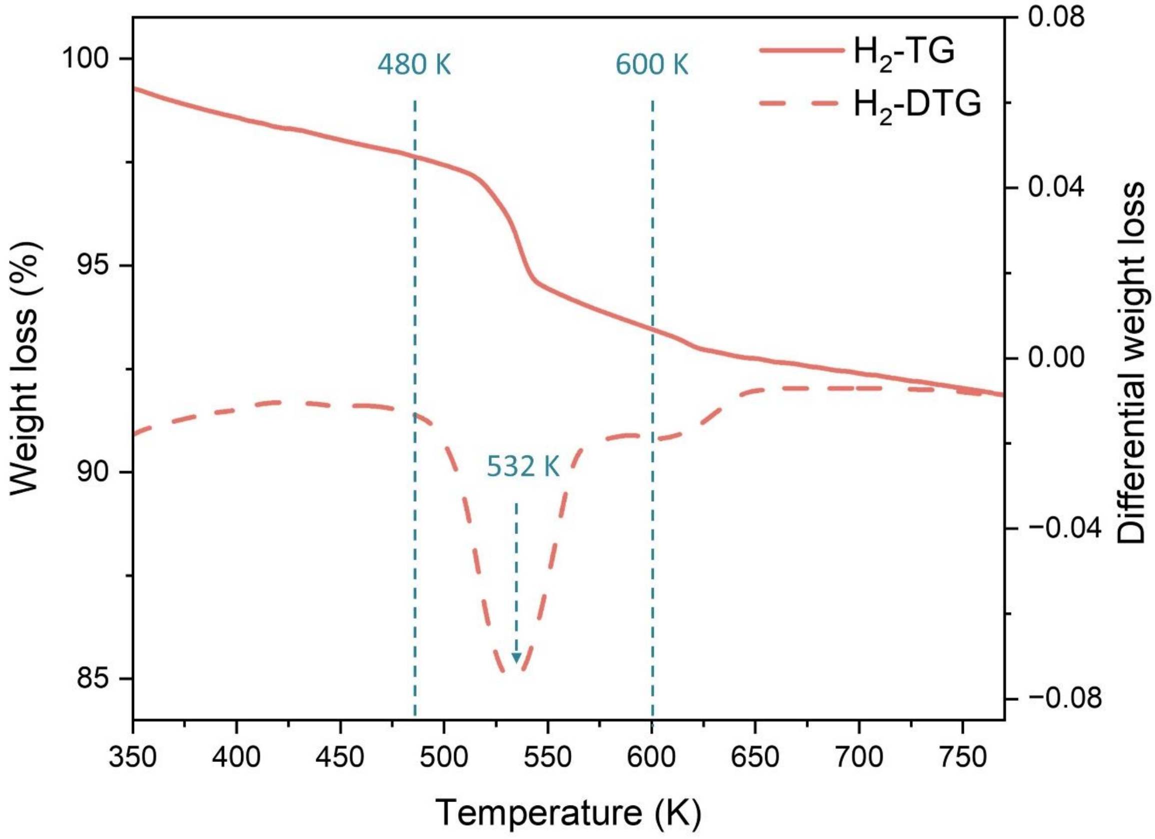

Figure S9. H_2-TGA and the corresponding differential TG curves of Cu_3-meIm-Z.

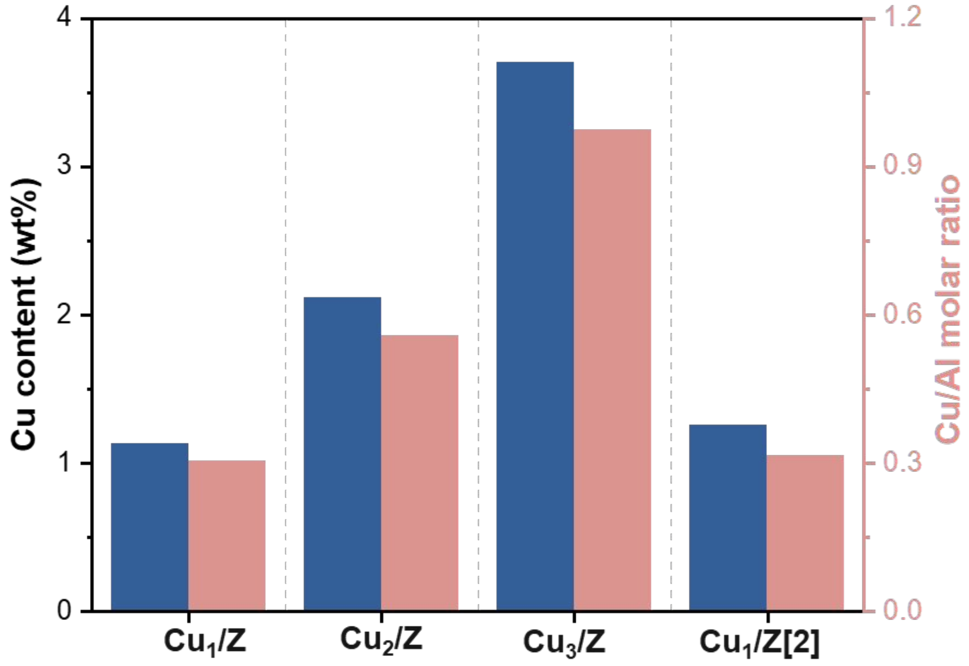

Figure S10. Copper content and the Cu: Al molar ratio of Cu_x/Z (x = 1, 2, and 3), and Cu_1/Z[2] ('Cu_1/Z[2]': ion-exchange with Cu ions for two times without ligand molecules).

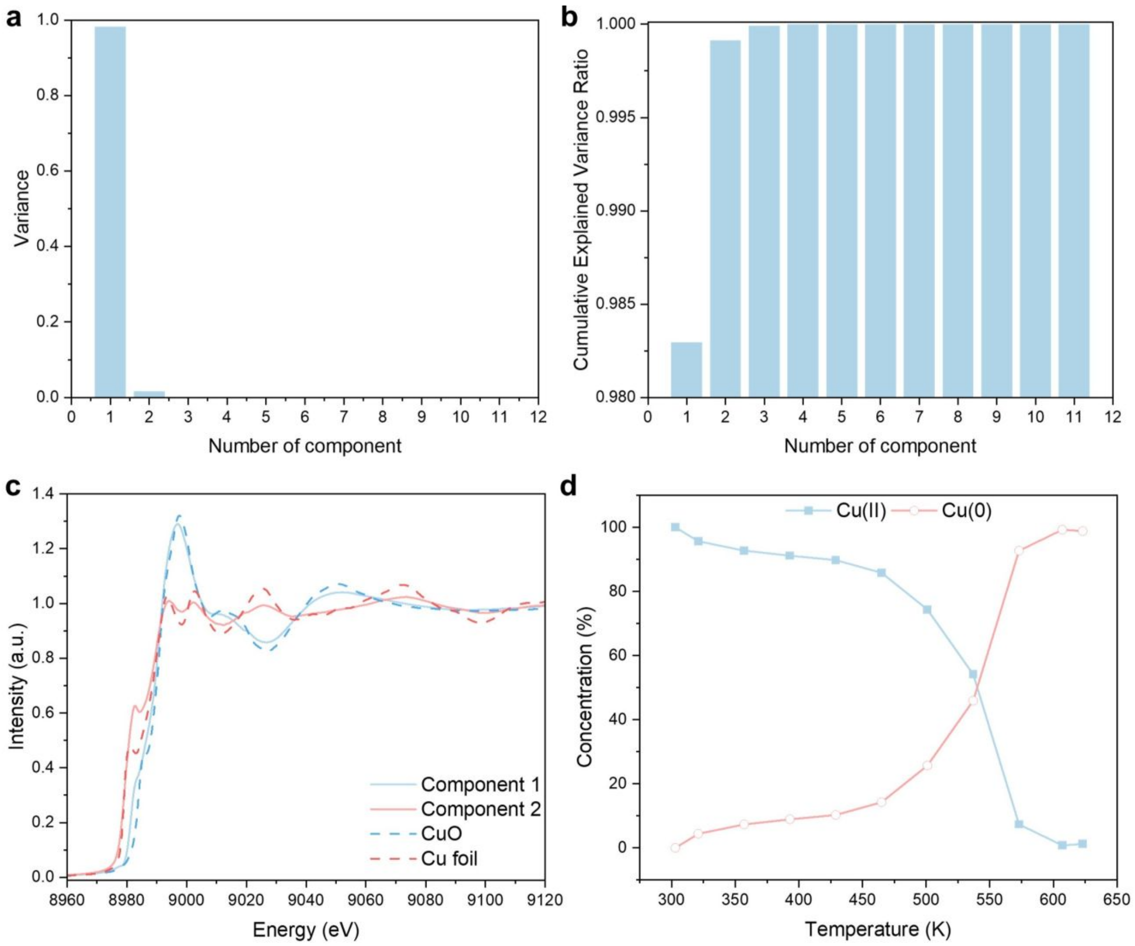

Figure S11. (**a**) PCA scree plot and (**b**) cumulative explained variance ratio showing the result from the *in situ* XANES result, (**c**) retrieved XANES spectra for the *in situ* XAS dataset collected from the reduction process of Cu_3-meIm-Z using the MCR-alternating least-squares (MCR-ALS) method, and (**d**) evolution of the relative amount of each component.

To further investigate the electronic structure of Cu_3/Z, principal component analysis (PCA) was initially applied to the in situ XANES data of Cu_3-meIm-Z (**Figure S11a − b**). The PCA results revealed that two principal components sufficiently accounted for all 11 experimental datasets. Furthermore, the multivariate curve resolution by alternating least-squares (MCR-ALS) analysis has been conducted to monitor the dynamic evolution of the electronic structure of copper species (**Figure S11c − d**). Two spectra can be retrieved from the *in situ* XANES data, in which the characteristics of component 1 are close to that of CuO, and another is close to that of Cu foil. At the temperature of 573 K, the Cu(0): Cu(II) ratio is 0.927: 0.073, indicating a mixed $Cu^{\delta+}$ state ($0 < \delta < 2$) of Cu_3/Z. The MCR-ALS result indicates the overall valence state of Cu_3/Z is close to +0.5. It should be noted that the difference in the overall valence state obtained from the XPS and MCR-ALS on the XAS is acceptable, as these techniques probe different aspects of the material and rely on distinct physical principles.

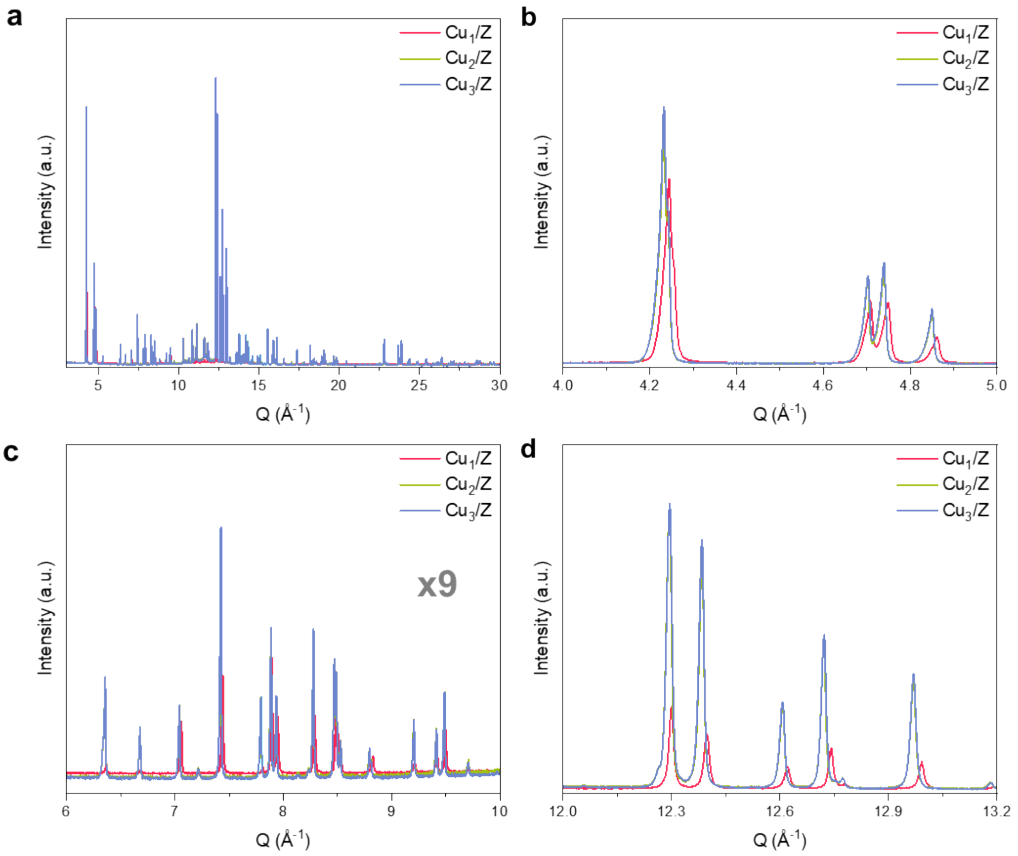

Figure S12. (**a**) Synchrotron PXRD data of Cu_x/Z, and (**b − d**) detailed comparison of the synchrotron PXRD patterns of the samples.

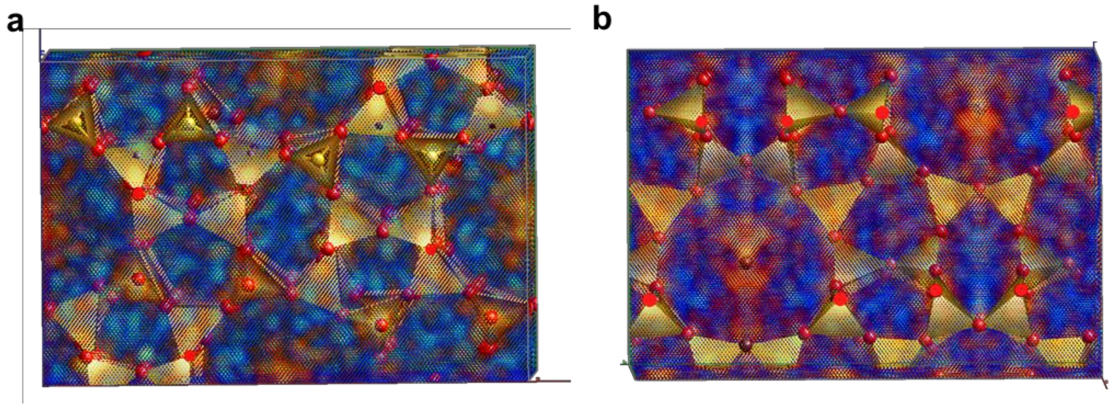

Figure S13. Fourier difference map derived by charge flipping (by TOPAS-v7.0) that determines the locations of the heavier atoms along the (**a**) [010], and (**b**) [100] zone axes.

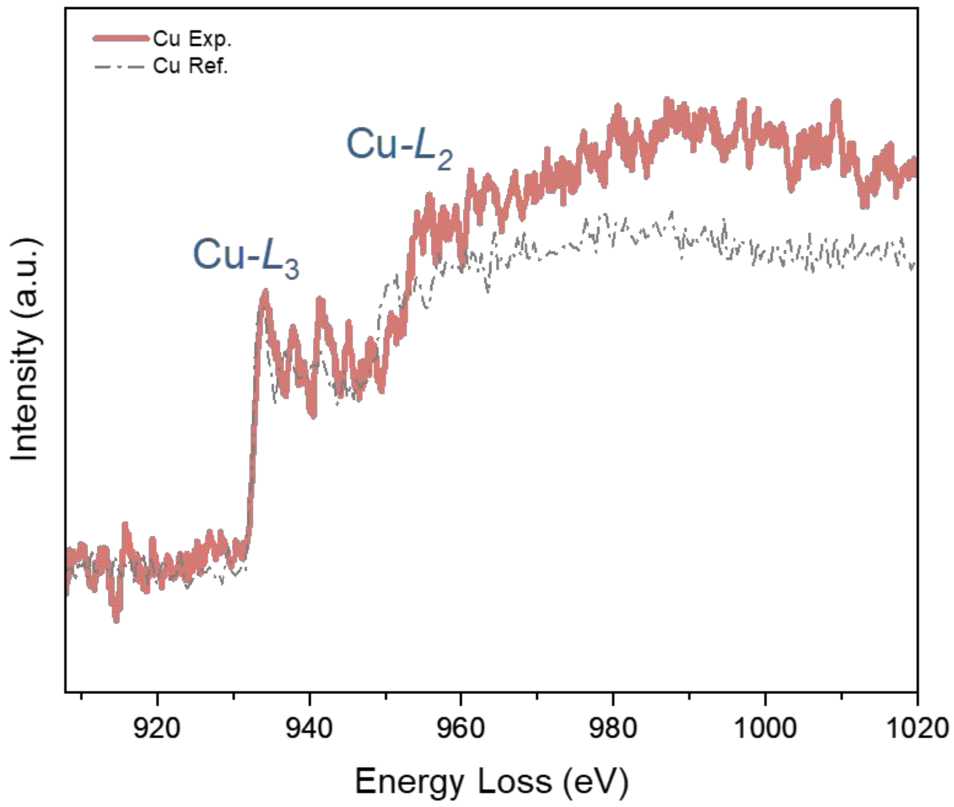

Figure S14. High-loss EELS fine structure of copper intercalated into the Cu_3/Z.

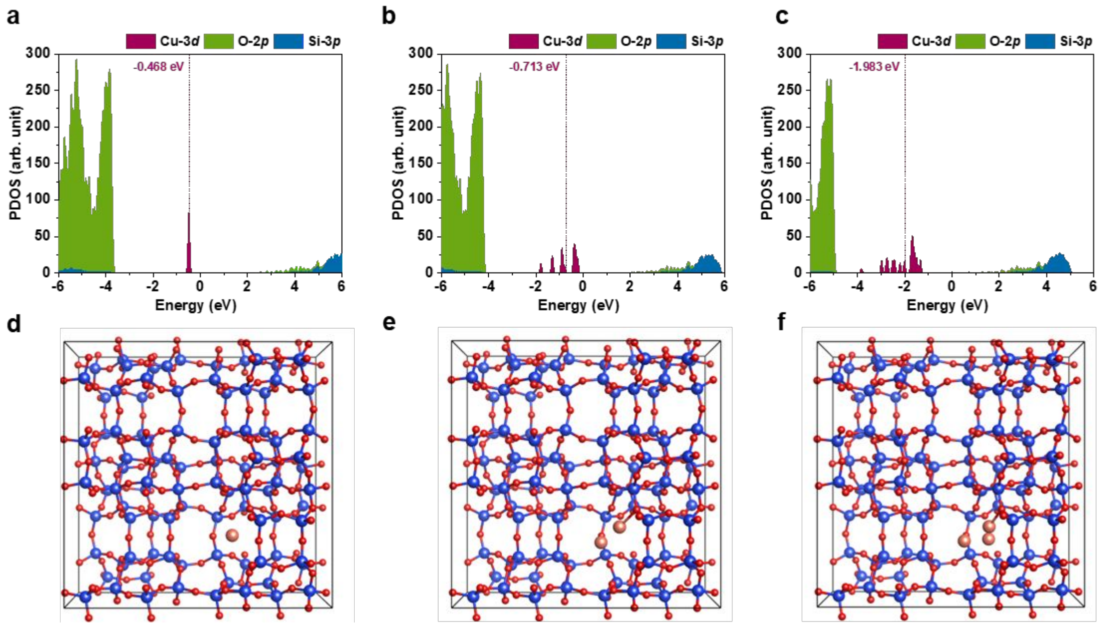

Figure S15. The projected density of states (PDOS) of the reduced (**a**) Cu_1/Z, (**b**) Cu_2/Z, (**c**) Cu_3/Z, and (**d** − **f**) the corresponding crystal structures. (blue = Si, red =O, and orange = Cu).

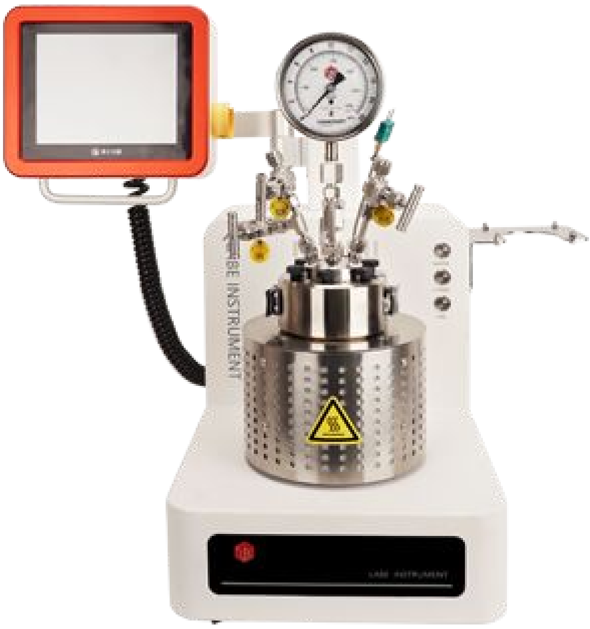

Figure S16. Photograph of the batch reactor (BE100 reactor, Shanghai LABE Instrument Co,. Ltd.).

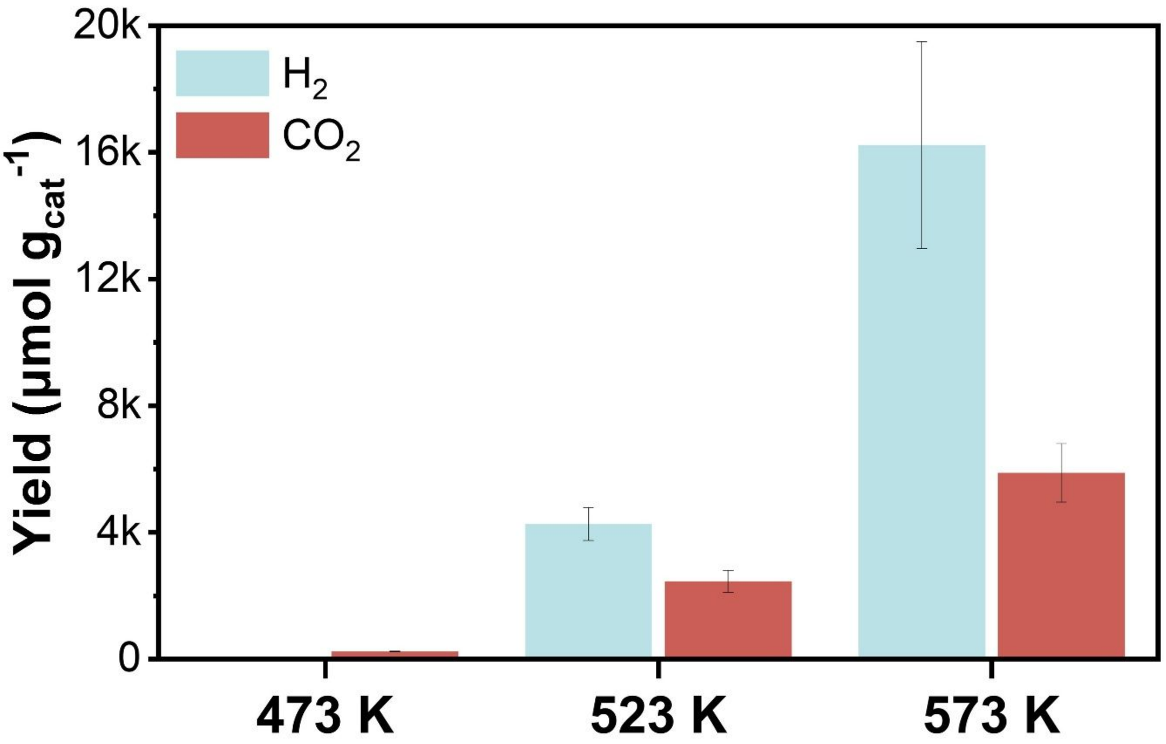

Figure S17. Catalytic performance evaluation of Cu_3/Z at different reaction temperatures, with a methanol-to-water ratio of 1:1.

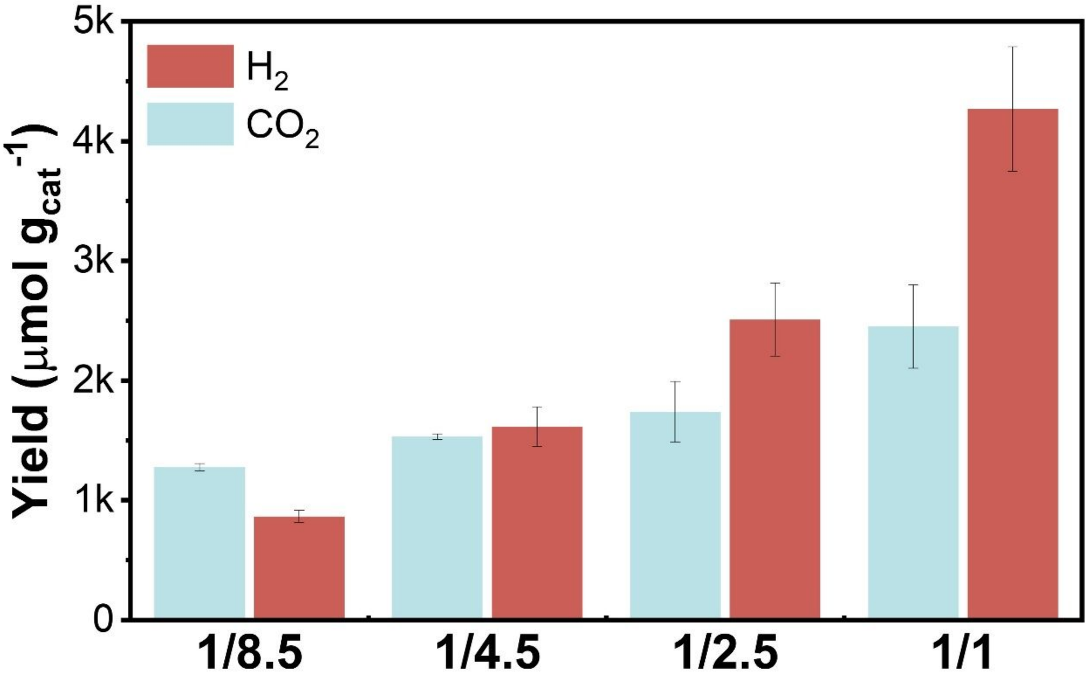

Figure S18. Catalytic performance evaluation of Cu_3/Z at different methanol-to-water ratios. Reaction temperature = 523 K.

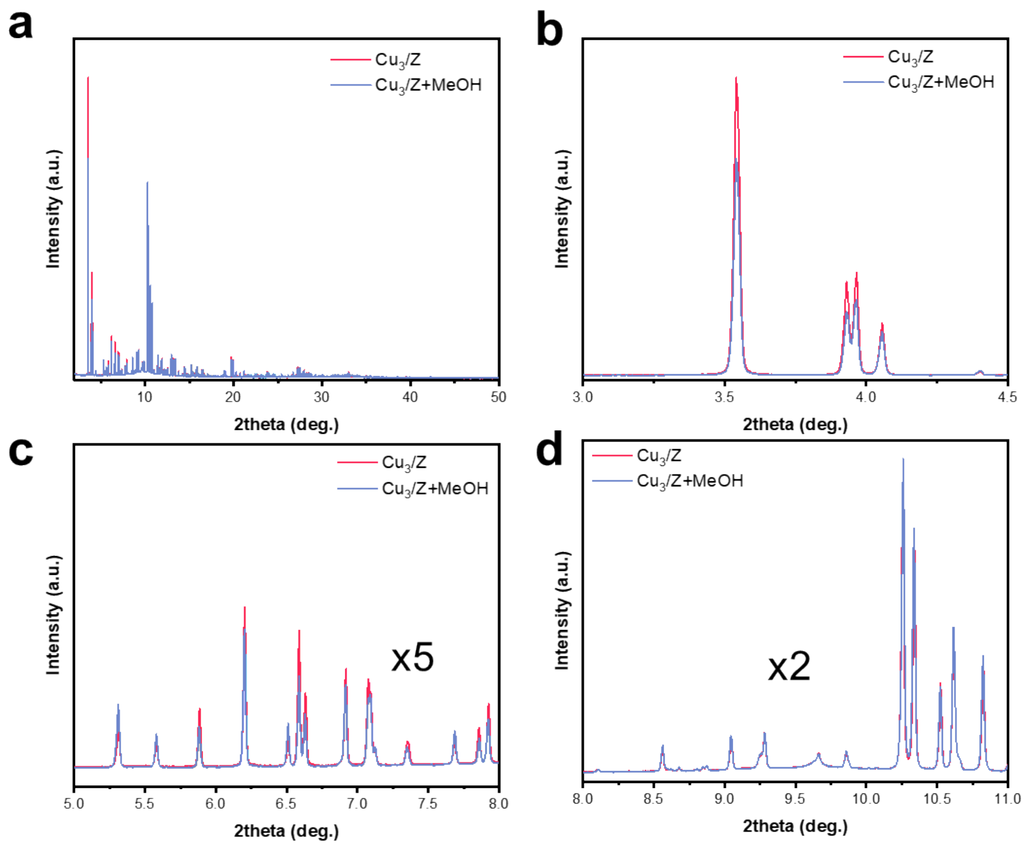

Figure S19. (**a**) Synchrotron PXRD data of Cu_3/Z and Cu_3/Z pre-adsorbed with methanol (Cu_3/Z+MeOH) (E = 18 keV, λ = 0.688657 (2) Å), and (**b − d**) comparison of the diffraction patterns of the samples in detail.

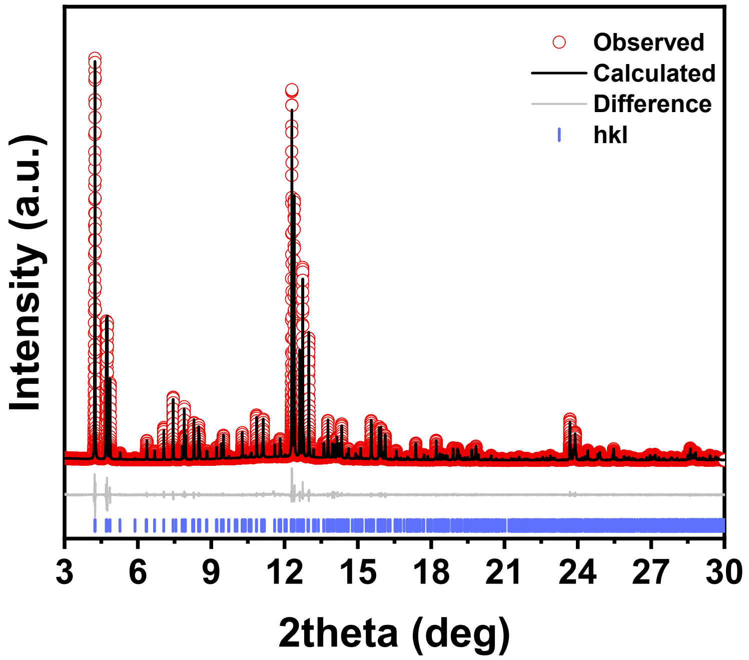

Figure S20. Synchrotron PXRD patterns and the Rietveld refinement profile of Cu_1/Z+MeOH.

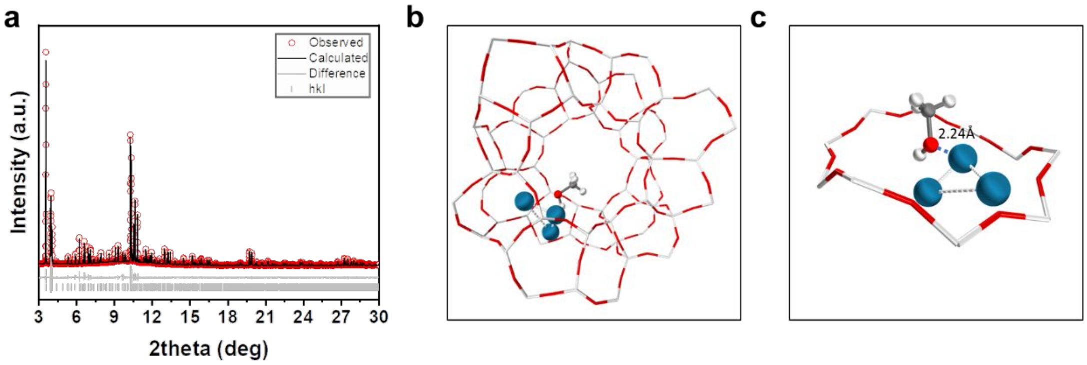

Figure S21. Synchrotron PXRD patterns and the Rietveld refinement profile of Cu_3/Z (Site B) pre-adsorbed with methanol.

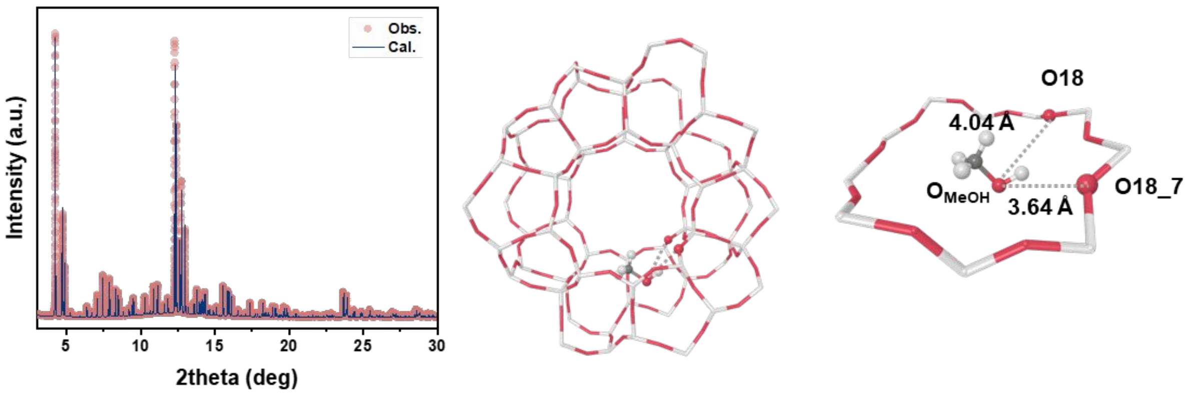

Figure S22. Synchrotron PXRD patterns and the Rietveld refinement profile of pristine ZSM-5 pre-adsorbed with methanol.

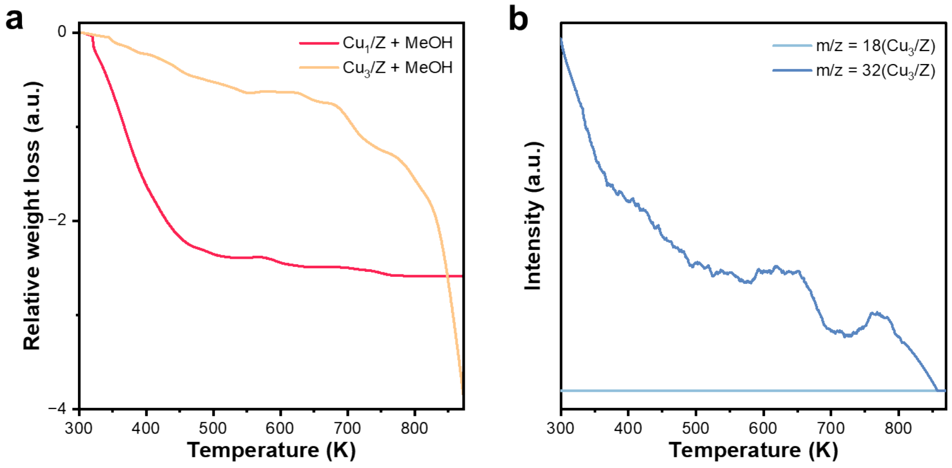

Figure S23. TG-MS curves of Cu_1/Z and Cu_3/Z with pre-adsorbed methanol.

Supplementary Tables

Table S1. Coordination environments derived from *in-situ* X-ray absorption spectroscopy; quantitative fitting parameters of Cu_3-meIm-Z. CN is the average coordination number around the central atoms. R and σ^2 are the average bond distance and the Debye-Waller factor, respectively. The ΔE_0 values of two Cu–X shells are constrained to share the same value in the fitting models.

Temp. (K)	Path	CN	R/Å	σ^2/Å^2	ΔE	R-factor	k-range/Å^{-1}
303	Cu-N	2.9(1)	1.959(6)	0.003(1)	3.7(3)	0.014	3-12
	Cu-C	1.4(3)	2.930(19)	0.002(1)			
321	Cu-N	2.8(2)	1.953(6)	0.003(1)	4.0(8)	0.008	3-12
	Cu-C	0.9(2)	2.882(26)	0.002(1)			
357	Cu-N	2.7(1)	1.958(4)	0.004(1)	3.0(2)	0.005	3-12
	Cu-C	1.1(1)	2.940(12)	0.002(1)			
393	Cu-N	2.7(1)	1.959(4)	0.004(1)	2.9(2)	0.005	3-12
	Cu-C	0.9(2)	2.933(18)	0.003(1)		0.005	
429	Cu-N	3.4(2)	1.937(12)	0.006(2)	7.5(5)	0.019	3-12
465	Cu-N	3.0(1)	1.962(14)	0.008(4)	3.7(4)	0.007	3-10
501	Cu-N	2.7(1)	1.956(7)	0.006(1)	4.4(3)	0.003	3-12
537	Cu-N	2.1(1)	1.917(10)	0.005(2)	2.8(5)	0.001	3-12
573	Cu-N	1.1(1)	1.889(16)	0.009(6)	1.4(7)	0.020	3-12
	Cu-Cu	2.0(1)	2.522(15)	0.009(1)			
601	Cu-Cu	5.6(3)	2.512(9)	0.013(1)	0.9(5)	0.019	3-12
623	Cu-Cu	6.2(3)	2.520(8)	0.013(1)	1.4(4)	0.018	3-12

s_0^2 was set at 0.98. It is worth noting that the backscattering by Cu–O and Cu–N bonds are nearly indistinguishable by EXAFS due to the proximity of their scattering factors.

Table S2. Coordination environments derived from *in-situ* X-ray absorption spectroscopy; quantitative fitting parameters of Cu_2-meIm-Z. CN is the average coordination number around the central atoms. R and σ^2 are the average bond distance and the Debye-Waller factor, respectively. The ΔE_0 values of two Cu–X shells are constrained to share the same value in the fitting models.

**Temp. (K)**	**Path**	**CN**	**R/Å**	**σ^2/Å^2**	**ΔE**	**R-factor**	**k-range/Å^{-1}**
303	Cu-N	3.5(2)	1.946(11)	0.006(1)	-6.7(6)	0.015	3-10
321	Cu-N	3.2(2)	1.955(13)	0.005(2)	-3.8(6)	0.015	3-9.5
	Cu-C	2.3(5)	3.129(24)	0.006(3)			
357	Cu-N	2.7(1)	1.945(8)	0.003(1)	-5.8(4)	0.006	3-10
393	Cu-N	2.7(1)	1.962(7)	0.004(1)	-4.3(4)	0.007	3-10
429	Cu-N	2.6(1)	1.968(13)	0.003(1)	-3.9(6)	0.011	3-9
465	Cu-N	2.6(2)	1.954(18)	0.005(1)	-6.0(7)	0.011	3-9
501	Cu-N	1.9(1)	1.960(14)	0.003(1)	6.0(6)	0.012	3-9.5
537	Cu-N	2.0(1)	1.935(12)	0.005(2)	5.4(5)	0.020	3-10
573	Cu-N	1.0(1)	1.900(16)	0.003(1)	5.7(10)	0.020	3-9.4
	Cu-Cu	1.4(1)	2.559(18)	0.003(1)			
607	Cu-Cu	4.8(2)	2.512(9)	0.011(2)	0.3(5)	0.010	3-11
623	Cu-Cu	6.6(4)	2.512(13)	0.014(3)	0.0(6)	0.018	3-11

s_0^2 was set at 0.9. It is worth noting that the backscattering by Cu–O and Cu–N bonds are nearly indistinguishable by EXAFS due to the proximity of their scattering factors.

Table S3. The fitting result in Cu $2p_{3/2}$ and Cu LMM Auger electron spectra of Cu_x/Z.

Name	Spectra	Position	FWHM	Area	ratio	Auger parameter α
Cu_1/Z	Cu $2p_{3/2}$	932.30	1.17	6599.65	58.7%	1851.42
		932.99	3.33	4647.24	41.3%	
	Cu LMM	919.12	1.26	983.12	90.2%	
		918.61	1.42	107.22	9.8%	
Cu_2/Z	Cu $2p_{3/2}$	932.08	1.48	9266.03	48.1%	1851.43
		932.83	2.82	9996.08	51.9%	
	Cu LMM	919.35	1.19	1133.20	70.8%	
		918.52	1.19	467.02	29.2%	
Cu_3/Z	Cu $2p_{3/2}$	931.96	1.21	9790.75	50.5%	1851.33
		932.55	2.98	9606.34	49.5%	
	Cu LMM	919.37	1.17	1448.15	69.1%	
		918.68	1.17	647.22	30.9%	

Table S4. Elemental analysis of Cu_x/Z by ICP-OES.

Sample	Element concentration (wt%)		
	Si	Al	Cu
Cu_1/Z	35.9336	1.5142	1.13
Cu_2/Z	33.7485	1.608	2.12
Cu_3/Z	32.2777	1.6151	3.70

Table S5. Crystallographic parameters of Cu_x/Z from the Rietveld refinement of synchrotron PXRD data.

	**Cu_1/Z**	**Cu_2/Z**	**Cu_3/Z**
X-ray energy (keV)	15	15	15
Beamline	Diamond I11	Diamond I11	Diamond I11
Wavelength (Å) (synchrotron)	0.825838(2)	0.824681(2)	0.824681(2)
2θ - zero point (°)	0.0092	0.0012	0.0012
Space group	*Pnma*	*Pnma*	*Pnma*
Crystal system	Orthorhombic	Orthorhombic	Orthorhombic
a (Å)	20.1322(3)	20.0969(5)	20.0950(5)
b (Å)	19.9591(3)	19.9377(5)	19.9381(4)
c (Å)	13.4268(3)	13.5282(4)	13.4274(4)
V (Å^3)	5395.2(2)	5380.5(2)	5379.7(2)
2θ range for refinement (°)	3-55	3-55	3-55
Number of parameters	47	46	52
Number of *hkl*s	4186	4183	4190
Refinement methods	Rietveld	Rietveld	Rietveld
$R_{wp}/R_{exp}/R_p$ (%)	8.014/6.682/6.280	11.596/3.961/8.757	12.211/3.238/8.749
Gof	1.168	2.927	3.771

R_{wp}: weighted profile; R_{exp}: expected; R_p: profile; *gof:* goodness-of-fit.

Table S6. Atomic parameters of Cu_1/Z from the Rietveld refinement of synchrotron PXRD data.

Species	Atom	x	y	z	SOF	Beq	Wyckoff
Zeolite framework	O1	0.3818	0.0481	0.7417	1	2.3074	8d
	O2	0.3068	0.0591	0.9267	1	2.3074	8d
	O3	0.1977	0.0627	0.0221	1	2.3074	8d
	O4	0.0967	0.0654	0.9125	1	2.3074	8d
	O5	0.1134	0.0523	0.7303	1	2.3074	8d
	O6	0.2467	0.0517	0.7504	1	2.3074	8d
	O7	0.3744	0.8550	0.7579	1	2.3074	8d
	O8	0.3078	0.8435	0.9316	1	2.3074	8d
	O9	0.1889	0.8474	0.0308	1	2.3074	8d
	O10	0.0863	0.8389	0.9242	1	2.3074	8d
	O11	0.1219	0.8403	0.7369	1	2.3074	8d
	O12	0.2439	0.8422	0.7644	1	2.3074	8d
	O13	0.3098	0.9455	0.8185	1	2.3074	8d
	O14	0.0750	0.9525	0.8283	1	2.3074	8d
	O15	0.4222	0.1331	0.6092	1	2.3074	8d
	O16	0.4072	1.0017	0.5850	1	2.3074	8d
	O17	0.4026	0.8649	0.5714	1	2.3074	8d
	O18	0.1918	0.1286	0.6142	1	2.3074	8d
	O19	0.2004	-0.0010	0.5962	1	2.3074	8d
	O20	0.2037	0.8682	0.5813	1	2.3074	8d
	O21	0.9976	0.0500	0.7989	1	2.3074	8d
	O22	0.9984	0.8474	0.7954	1	2.3074	8d
	O23	0.4198	0.7500	0.6384	1	2.3074	4c
	O24	0.1966	0.7500	0.6567	1	2.3074	4c
	O25	0.2821	0.7500	0.0591	1	2.3074	4c
	O26	0.0973	0.7500	0.0631	1	2.3074	4c

	Si1	0.4225	0.0576	0.6582	1	1.1537	8d
	Si2	0.3078	0.0302	0.8166	1	1.1537	8d
	Si3	0.2786	0.0601	0.0361	1	1.1537	8d
	Si4	0.1197	0.0641	0.0283	1	1.1537	8d
	Si5	0.0703	0.0299	0.8147	1	1.1537	8d
	Si6	0.1867	0.0564	0.6754	1	1.1537	8d
	Si7	0.4255	0.8288	0.6748	1	1.1537	8d
	Si8	0.3090	0.8702	0.8168	1	1.1537	8d
	Si9	0.2749	0.8275	0.0323	1	1.1537	8d
	Si10	0.1225	0.8246	0.0298	1	1.1537	8d
	Si11	0.0713	0.8711	0.8206	1	1.1537	8d
	Si12	0.1890	0.8272	0.6824	1	1.1537	8d
Mononuclear Cu_1 site	Cu	0.6158	0.2030	0.7418	0.28	5	8d

a: Based on the elemental analysis of Cu_1/Z from ICP-OES, the SOF of the mononuclear Cu_1 site was constrained at 0.28.

Table S7. Atomic parameters of Cu_2/Z from the Rietveld refinement of synchrotron PXRD data.

Species	Atom	x	y	z	SOF	Beq	Wyckoff
Zeolite framework	O1	0.3683	0.0546	0.7491	1	0.290	8d
	O2	0.3119	0.0632	0.9145	1	0.290	8d
	O3	0.2002	0.0566	0.0270	1	0.290	8d
	O4	0.1006	0.0599	0.9114	1	0.290	8d
	O5	0.1097	0.0509	0.7211	1	0.290	8d
	O6	0.2429	0.0523	0.7422	1	0.290	8d
	O7	0.3724	0.8482	0.7955	1	0.290	8d
	O8	0.2902	0.8460	0.9246	1	0.290	8d
	O9	0.2007	0.8439	0.0146	1	0.290	8d
	O10	0.0913	0.8343	0.9167	1	0.290	8d
	O11	0.1124	0.8475	0.7364	1	0.290	8d
	O12	0.2240	0.8368	0.7166	1	0.290	8d
	O13	0.3002	0.9568	0.8116	1	0.290	8d
	O14	0.0806	0.9478	0.8144	1	0.290	8d
	O15	0.4168	0.1314	0.6093	1	0.290	8d
	O16	0.4189	0.9974	0.5784	1	0.290	8d
	O17	0.4035	0.8697	0.5773	1	0.290	8d
	O18	0.1776	0.1330	0.6148	1	0.290	8d
	O19	0.1980	0.0045	0.5976	1	0.290	8d
	O20	0.2121	0.8647	0.5629	1	0.290	8d
	O21	0.9966	0.0463	0.7976	1	0.290	8d
	O22	0.9993	0.8424	0.7686	1	0.290	8d
	O23	0.4106	0.7500	0.6052	1	0.290	4c
	O24	0.1899	0.7500	0.6545	1	0.290	4c
	O25	0.2974	0.7500	0.0540	1	0.290	4c
	O26	0.1049	0.7500	0.0705	1	0.290	4c

	Si1	0.4222	0.0557	0.6584	1	0.143	8d
	Si2	0.3063	0.0336	0.8087	1	0.143	8d
	Si3	0.2813	0.0611	0.0265	1	0.143	8d
	Si4	0.1202	0.0625	0.0291	1	0.143	8d
	Si5	0.0744	0.0295	0.8089	1	0.143	8d
	Si6	0.1829	0.0562	0.6636	1	0.143	8d
	Si7	0.4259	0.8313	0.6791	1	0.143	8d
	Si8	0.3093	0.8716	0.8128	1	0.143	8d
	Si9	0.2772	0.8269	0.0276	1	0.143	8d
	Si10	0.1202	0.8241	0.0202	1	0.143	8d
	Si11	0.0675	0.8678	0.8049	1	0.143	8d
	Si12	0.1863	0.8225	0.6700	1	0.143	8d
Binuclear Cu_2 site	Cu1	0.6404	0.2737	-0.2622	0.18	5	8d
	Cu2	0.5725	0.2268	-0.3467	0.18	5	8d

a: Based on the elemental analysis of Cu_2/Z from ICP-OES, the SOF of the binuclear Cu_2 site was constrained at 0.18 for each Cu atom.

Table S8. Atomic parameters of Cu_3/Z from the Rietveld refinement of synchrotron PXRD data.

Species	Atom	x	y	z	SOF	Beq	Wyckoff
Zeolite framework	O1	0.3681	0.0578	0.7502	1	1.0966	8d
	O2	0.3018	0.0626	0.9107	1	1.0966	8d
	O3	0.2008	0.0599	0.0368	1	1.0966	8d
	O4	0.0996	0.0629	0.9175	1	1.0966	8d
	O5	0.1161	0.0429	0.7261	1	1.0966	8d
	O6	0.2410	0.0581	0.7444	1	1.0966	8d
	O7	0.3871	0.8438	0.7979	1	1.0966	8d
	O8	0.3103	0.8333	0.9286	1	1.0966	8d
	O9	0.1991	0.8479	0.0044	1	1.0966	8d
	O10	0.0834	0.8394	0.9055	1	1.0966	8d
	O11	0.1240	0.8345	0.7307	1	1.0966	8d
	O12	0.2506	0.8514	0.7336	1	1.0966	8d
	O13	0.3036	0.9401	0.8245	1	1.0966	8d
	O14	0.0776	0.9546	0.8037	1	1.0966	8d
	O15	0.4171	0.1240	0.5984	1	1.0966	8d
	O16	0.4119	0.9847	0.5859	1	1.0966	8d
	O17	0.3999	0.8652	0.5756	1	1.0966	8d
	O18	0.1801	0.1314	0.6185	1	1.0966	8d
	O19	0.1985	0.0097	0.5895	1	1.0966	8d
	O20	0.2145	0.8560	0.5588	1	1.0966	8d
	O21	1.0017	0.0456	0.7958	1	1.0966	8d
	O22	0.9922	0.8416	0.7702	1	1.0966	8d
	O23	0.4280	0.7500	0.6221	1	1.0966	4c
	O24	0.1994	0.7500	0.6546	1	1.0966	4c
	O25	0.2823	0.7500	0.0652	1	1.0966	4c
	O26	0.1001	0.7500	0.0639	1	1.0966	4c

	Si1	0.4239	0.0514	0.6595	1	0.5483	8d
	Si2	0.3027	0.0271	0.8050	1	0.5483	8d
	Si3	0.2776	0.0597	0.0229	1	0.5483	8d
	Si4	0.1206	0.0626	0.0312	1	0.5483	8d
	Si5	0.0688	0.0338	0.8115	1	0.5483	8d
	Si6	0.1835	0.0592	0.6685	1	0.5483	8d
	Si7	0.4197	0.8280	0.6646	1	0.5483	8d
	Si8	0.3108	0.8705	0.8184	1	0.5483	8d
	Si9	0.2786	0.8278	0.0316	1	0.5483	8d
	Si10	0.1185	0.8258	0.0263	1	0.5483	8d
	Si11	0.0702	0.8736	0.8042	1	0.5483	8d
	Si12	0.1857	0.8279	0.6753	1	0.5483	8d
Trinuclear Cu_3 Site A	Cu1	0.4272	0.2927	-0.0996	0.16	5	8d
	Cu2	0.6580	0.2784	-0.2579	0.16	5	8d
	Cu3	0.4768	0.4129	-0.0519	0.16	5	8d
Trinuclear Cu_3 Site B	Cu4	0.5526	0.2301	-0.2756	0.16	5	8d
	Cu5	0.5322	0.1687	-0.0397	0.16	5	8d
	Cu6	0.5983	0.2640	-0.3726	0.16	5	8d

a: Based on the elemental analysis of Cu_3/Z from ICP-OES, the SOF of the trinuclear Cu_3 site was constrained at 0.16 for each Cu atom.

Table S9. Crystallographic parameters of Cu_1/Z and Cu_3/Z pre-adsorbed with methanol.

	**Cu_1/Z+MeOH**	**Cu_3/Z+MeOH**
X-ray energy (keV)	15	18
Beamline	Diamond I11	Spring-8 BL02B2
Wavelength (Å) (synchrotron)	0.825848(2)	0.688657(2)
2θ - zero point (°)	0.0092	0.0005
Space group	*Pnma*	*Pnma*
Crystal system	Orthorhombic	Orthorhombic
a (Å)	20.1321(1)	20.1033(5)
b (Å)	19.9592(1)	19.9430(2)
c (Å)	13.4268(1)	13.4303(1)
V ($Å^3$)	5395.2(1)	5384.46(9)
2θ range for refinement (°)	3-55	3-55
Number of parameters	33	37
Number of *hkl*s	4186	7177
Refinement methods	Rietveld	Rietveld
$R_{wp}/R_{exp}/R_p$ (%)	8.427/6.863/6.667	10.479/1.396/6.931
Gof	1.228	7.505

R_{wp}: weighted profile; R_{exp}: expected; R_p: profile; *gof:* goodness-of-fit.

Table S10. Atomic parameters of Cu_1/Z+MeOH from the Rietveld refinement of synchrotron PXRD data. The sample was dried at 100 °C overnight to remove physisorbed methanol species.

Species	Atom	x	y	z	SOF	Beq	Wyckoff
Zeolite framework	O1	0.3748	0.0646	0.7602	1	0.7893	8d
	O2	0.3077	0.0577	0.9219	1	0.7893	8d
	O3	0.2006	0.0633	0.0213	1	0.7893	8d
	O4	0.0889	0.0619	0.9125	1	0.7893	8d
	O5	0.1171	0.0484	0.7274	1	0.7893	8d
	O6	0.2482	0.0529	0.7495	1	0.7893	8d
	O7	0.3747	0.8530	0.7591	1	0.7893	8d
	O8	0.3103	0.8462	0.9317	1	0.7893	8d
	O9	0.1896	0.8486	0.0309	1	0.7893	8d
	O10	0.0869	0.8369	0.9289	1	0.7893	8d
	O11	0.1178	0.8430	0.7382	1	0.7893	8d
	O12	0.2452	0.8379	0.7650	1	0.7893	8d
	O13	0.3117	0.9478	0.8187	1	0.7893	8d
	O14	0.0766	0.9501	0.8307	1	0.7893	8d
	O15	0.4189	0.1333	0.6086	1	0.7893	8d
	O16	0.4155	1.0120	0.5772	1	0.7893	8d
	O17	0.4007	0.8700	0.5787	1	0.7893	8d
	O18	0.1947	0.1243	0.6064	1	0.7893	8d
	O19	0.1954	-0.0093	0.6092	1	0.7893	8d
	O20	0.2048	0.8667	0.5819	1	0.7893	8d
	O21	0.9964	0.0545	0.7926	1	0.7893	8d
	O22	0.9964	0.8503	0.7969	1	0.7893	8d
	O23	0.4198	0.7500	0.6393	1	0.7893	4c
	O24	0.1910	0.7500	0.6636	1	0.7893	4c
	O25	0.2853	0.7500	0.0683	1	0.7893	4c

	O26	0.1013	0.7500	0.0590	1	0.7893	4c
	Si1	0.4221	0.0565	0.6648	1	0.3947	8d
	Si2	0.3110	0.0297	0.8139	1	0.3947	8d
	Si3	0.2784	0.0594	0.0334	1	0.3947	8d
	Si4	0.1189	0.0637	0.0286	1	0.3947	8d
	Si5	0.0711	0.0291	0.8125	1	0.3947	8d
	Si6	0.1911	0.0582	0.6735	1	0.3947	8d
	Si7	0.4269	0.8291	0.6728	1	0.3947	8d
	Si8	0.3081	0.8703	0.8208	1	0.3947	8d
	Si9	0.2747	0.8268	0.0322	1	0.3947	8d
	Si10	0.1226	0.8248	0.0293	1	0.3947	8d
	Si11	0.0743	0.8694	0.8192	1	0.3947	8d
	Si12	0.1883	0.8269	0.6864	1	0.3947	8d
Mononuclear Cu_1 site	Cu	0.5986	0.2228	0.6885	0.28	5	8d
Adsorbed MeOH	C	0.0207	0.8200	0.4521	0.28	10	8d
	O	-0.0501	0.8265	0.4399	0.28	10	8d

Table S11. Atomic parameters of Cu_3/Z+MeOH from the Rietveld refinement of synchrotron PXRD data. The sample was dried at 100 °C overnight to remove physisorbed methanol species.

Species	Atom	x	y	z	SOF	Beq	Wyckoff
Zeolite framework	O1	0.3779	0.0312	0.7584	1	0.2180	8d
	O2	0.2993	0.0488	0.9162	1	0.2180	8d
	O3	0.2012	0.0432	0.0318	1	0.2180	8d
	O4	0.0854	0.0609	0.9171	1	0.2180	8d
	O5	0.1147	0.0618	0.7283	1	0.2180	8d
	O6	0.2473	0.0562	0.7462	1	0.2180	8d
	O7	0.3659	0.8370	0.7582	1	0.2180	8d
	O8	0.3065	0.8391	0.9235	1	0.2180	8d
	O9	0.2003	0.8418	0.0312	1	0.2180	8d
	O10	0.1015	0.8339	0.9069	1	0.2180	8d
	O11	0.1101	0.8491	0.7128	1	0.2180	8d
	O12	0.2343	0.8437	0.7632	1	0.2180	8d
	O13	0.3109	0.9511	0.8046	1	0.2180	8d
	O14	0.0861	0.9481	0.8278	1	0.2180	8d
	O15	0.4174	0.1334	0.6055	1	0.2180	8d
	O16	0.4208	0.9982	0.5777	1	0.2180	8d
	O17	0.3988	0.8730	0.5776	1	0.2180	8d
	O18	0.1946	0.1240	0.6118	1	0.2180	8d
	O19	0.1744	-0.0016	0.5827	1	0.2180	8d
	O20	0.1973	0.8664	0.5743	1	0.2180	8d
	O21	0.9968	0.0492	0.7973	1	0.2180	8d
	O22	1.0006	0.8477	0.7771	1	0.2180	8d
	O23	0.4262	0.7500	0.6513	1	0.2180	4c
	O24	0.2012	0.7500	0.6437	1	0.2180	4c
	O25	0.2839	0.7500	0.0643	1	0.2180	4c

	O26	0.1092	0.7500	0.0528	1	0.2180	4c
	Si1	0.4219	0.0586	0.6648	1	0.1090	8d
	Si2	0.3090	0.0302	0.8108	1	0.1090	8d
	Si3	0.2787	0.0604	0.0306	1	0.1090	8d
	Si4	0.1228	0.0629	0.0268	1	0.1090	8d
	Si5	0.0707	0.0305	0.8116	1	0.1090	8d
	Si6	0.1843	0.0567	0.6638	1	0.1090	8d
	Si7	0.4242	0.8267	0.6647	1	0.1090	8d
	Si8	0.3049	0.8706	0.8077	1	0.1090	8d
	Si9	0.2747	0.8256	0.0295	1	0.1090	8d
	Si10	0.1163	0.8278	0.0218	1	0.1090	8d
	Si11	0.0724	0.8694	0.8169	1	0.1090	8d
	Si12	0.1864	0.8278	0.6827	1	0.1090	8d
Trinuclear Cu_3 Site A	Cu1	-0.0068	0.8124	0.5369	0.16	5	8d
	Cu2	0.0749	0.8000	0.4239	0.16	5	8d
	Cu3	0.0207	0.9242	0.4963	0.16	5	8d
Adsorbed MeOH-A	C	-0.0181	0.7539	0.2865	0.16	10	8d
	O	-0.0187	0.7429	0.3924	0.16	10	8d
Trinuclear Cu_3 Site B	Cu4	-0.1092	0.8469	0.2213	0.16	5	8d
	Cu5	-0.1786	0.7774	0.1401	0.16	5	8d
	Cu6	-0.0204	0.8450	0.1063	0.16	5	8d
Adsorbed MeOH-B	C	-0.0510	0.7514	0.3683	0.16	10	8d
	O	-0.0628	0.7487	0.2626	0.16	10	8d

Supplementary References

1. Kawaguchi, S. *et al.* High-throughput powder diffraction measurement system consisting of multiple MYTHEN detectors at beamline BL02B2 of SPring-8. *Rev. Sci. Instrum.* **88**, 85111 (2017).

2. Scardi, P., Azanza Ricardo, C. L., Perez-Demydenko, C. & Coelho, A. A. Whole powder pattern modelling macros for TOPAS. *J. Appl. Crystallogr.* **51**, 1752–1765 (2018).

3. Thompson, P., Cox, D. E. & Hastings, J. B. Rietveld refinement of Debye–Scherrer synchrotron X-ray data from Al2O3. *J. Appl. Crystallogr.* **20**, 79–83 (1987).

4. Teixeira, I. F. *et al.* From Biomass-Derived Furans to Aromatics with Ethanol over Zeolite. *Angew. Chem. Int. Ed.* **55**, 13061–13066 (2016).

5. Chen, T. *et al.* Differential Adsorption of l- and d-Lysine on Achiral MFI Zeolites as Determined by Synchrotron X-Ray Powder Diffraction and Thermogravimetric Analysis. *Angew. Chem. Int. Ed.* **59**, 1093–1097 (2020).

6. Lo, B. T. W. *et al.* Elucidation of Adsorbate Structures and Interactions on Brønsted Acid Sites in H-ZSM-5 by Synchrotron X-ray Powder Diffraction. *Angew. Chem. Int. Ed.* **55**, 5981–5984 (2016).

7. Ravel, B. & Newville, M. ATHENA, ARTEMIS, HEPHAESTUS: Data analysis for X-ray absorption spectroscopy using IFEFFIT. in *Journal of Synchrotron Radiation* vol. 12 537–541 (International Union of Crystallography, 2005).

8. Funke, H., Scheinost, A. C. & Chukalina, M. Wavelet analysis of extended x-ray absorption fine structure data. *Phys. Rev. B - Condens. Matter Mater. Phys.* **71**, 94110 (2005).

9. Lonyi, F. & Valyon, J. On the interpretation of the NH3-TPD patterns of H-ZSM-5 and H-mordenite. *Microporous Mesoporous Mater.* **47**, 293–301 (2001).
